# Supplementary material for: Loss of the batten disease protein CLN3 leads to mis-trafficking of M6PR and defective autophagic-lysosomal reformation
Source: Nat Commun. 2023 Jul 3;14:3911. doi: 10.1038/s41467-023-39643-7 (PMC10317969; doi:10.1038/s41467-023-39643-7)
Supplement: Supplementary file 1 — Supplementary Information [file 41467_2023_39643_MOESM1_ESM.pdf]

Supplementary Fig.1

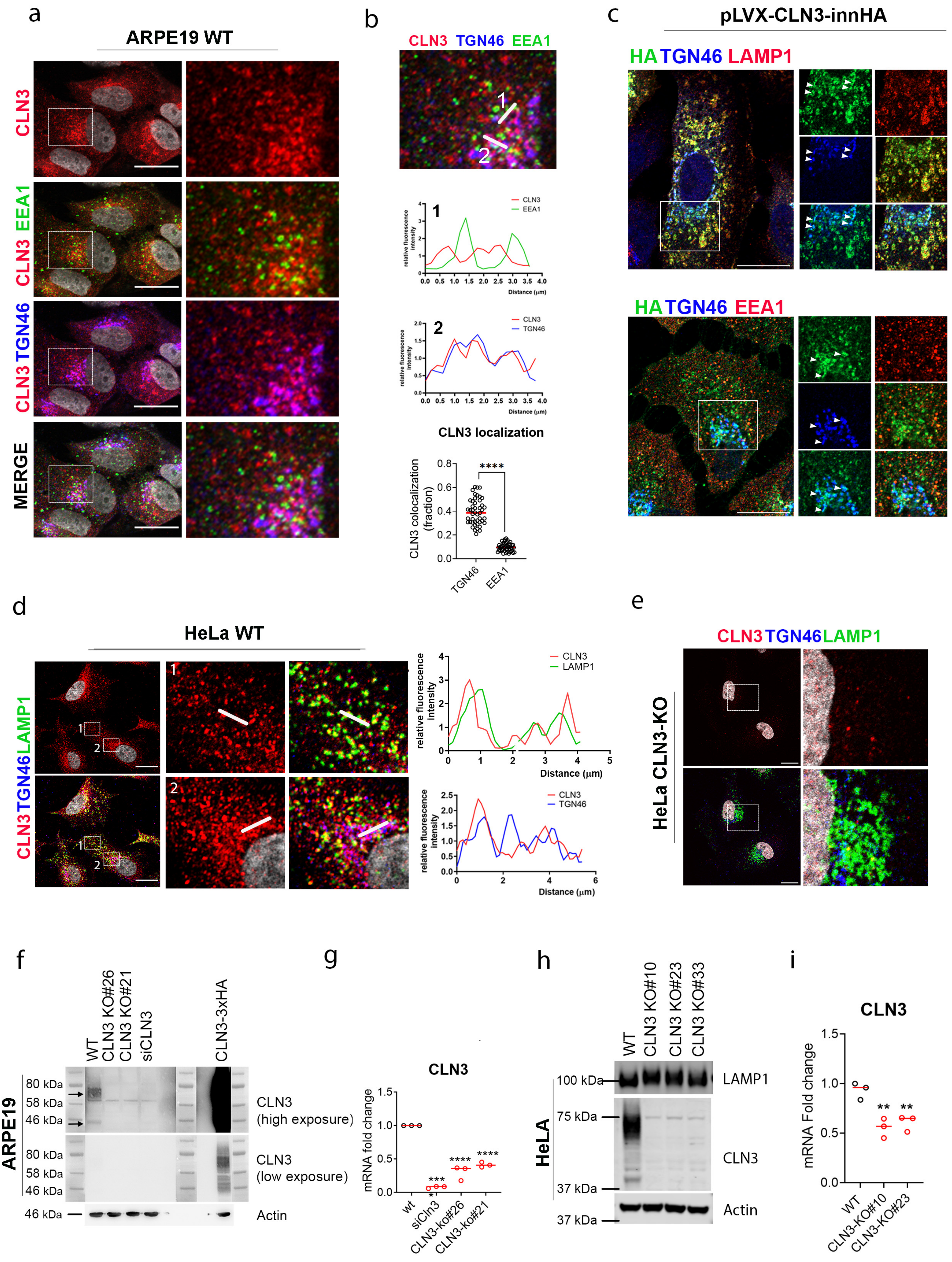

Supplementary Fig. 1 CLN3 protein localization

a, ARPE19 WT cells stained with antibodies against CLN3 (red), TGN46 (blue) and EEA1 (green), analyzed by confocal microscopy. Scale bar 20µm. **b,** Enlargements of **a** and fluorescent line intensity plot showing CLN3-EEA1 (1) and CLN3-TGN46 (2) co-localization. White lines show analyzed areas. CLN3-TGN46 and CLN3-EEA1 Manders' colocalization coefficients are shown. Results are means+single values. N=46 cells, P-value<0.0001, unpaired t test (two-tailed). **c,** pLVX CLN3-innHA ARPE19 cells**,** stained with the indicated antibodies, and analyzed by confocal microscopy. Scale bar 20µm. Insets show image enlargements. Fluorescent line intensity plots show CLN3-LAMP1 (1) and CLN3-TGN46 (2) co-localization. **d,** HeLa cells stained for the indicated antibodies and analyzed by confocal microscopy. Scale bar 20µm. Fluorescent line intensity plots show CLN3-LAMP1 (1) and CLN3-TGN46 (2) co-localization. **e,** CLN3-KO HeLa cells stained with the indicated antibodies. Scale bar 20µm . **f,** Immunoblot analysis showing absence of CLN3 protein bands in ARPE19 CLN3-depleted cells. Clones carry a premature stop codon (c.109delA). **g,** Real-time PCR showing significantly reduced CLN3 transcript levels in ARPE19 silenced and KO cells. N= three independent experiments, one-way Anova (****p<0.0001). **h,** Immunoblot validation of CLN3 depletion in HeLA CLN3 KO clones. **i,** Real-time PCR showing significantly reduced CLN3 transcript levels in HeLa CLN3-KO cells. N= three independent experiments, one-way Anova (**p<0.01). Source data are provided as a Source Data file.

Supplementary Fig.2

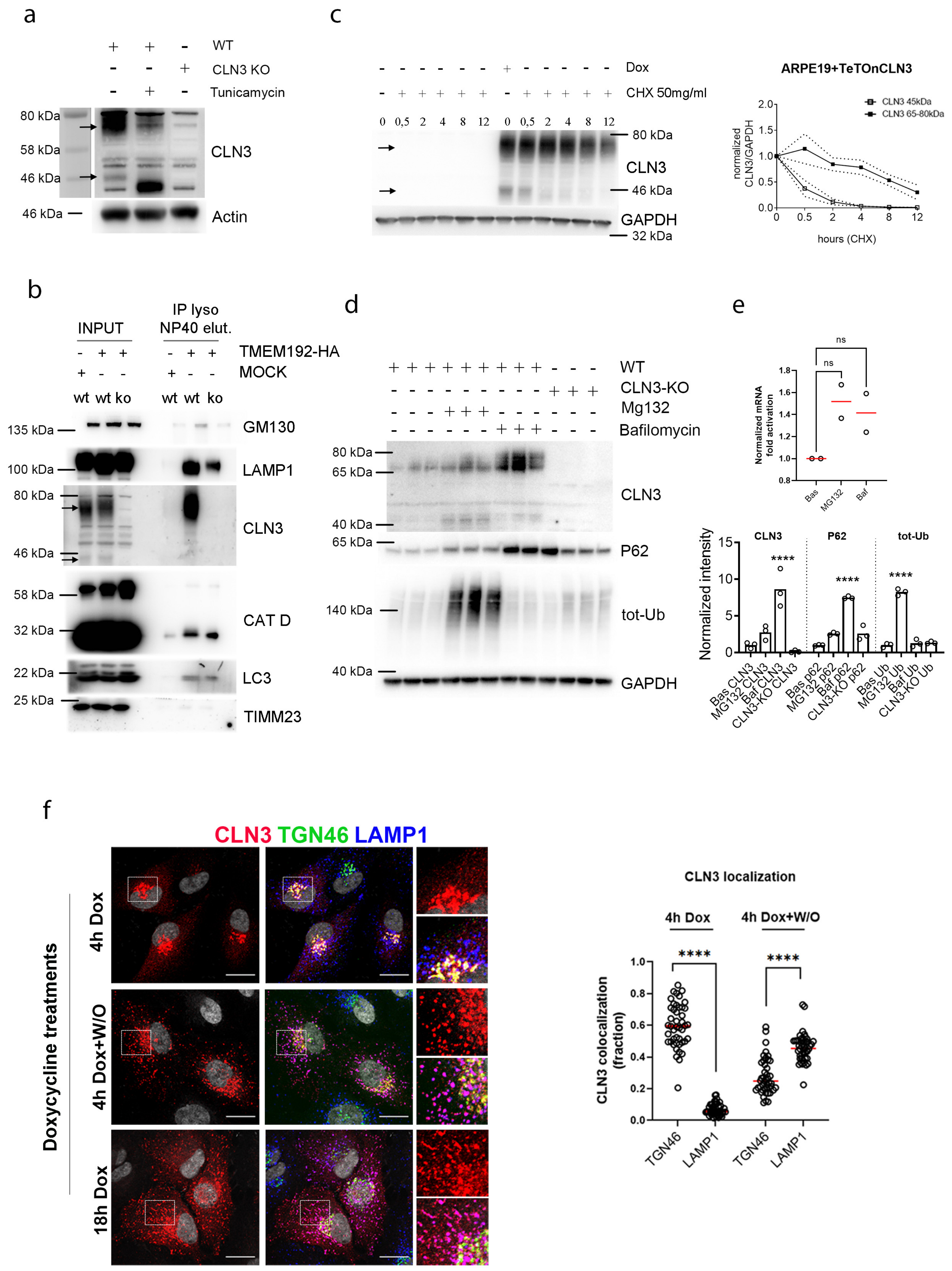

Supplementary Fig. 2 CLN3 protein maturation and degradation.
a, Immunoblot analysis of ARPE19 cells treated with 2 µg/mL tunicamycin for 16h and analyzed for the indicated antibodies. Repeated two times. **b,** Glycosylated CLN3 localizes to lysosomes. ARPE19 WT and CLN3 KO cells were infected with lenti-TMEM192-HA, and lysosomes were then immuno-purified with magnetic HA beads (Dynabeads) and subjected to immunoblot for the indicated antibodies. Repeated two times. **c,** Immunoblot analysis of CLN3 protein in ARPE19-pLVX-CLN3-*inn*HA cells induced with 1µg/ml doxycycline for 4h, and then washed and treated with 50µg/ml CHX to block protein synthesis for the indicated time points. Graph shows protein degradation rate of the two CLN3 molecular weight bands, values are normalized against GAPDH and T0 (mean+SEM). Repeated three times. **d,** Immunoblot analysis and relative quantification (mean+single values) of ARPE19 WT and CLN3 KO cells treated for 24h with 50µm MG132 or 20nM bafilomycin A1. Each lane is an independent replicate. N=three independent experiments, two-way Anova (****p<0.0001). **e,** ARPE19 cells were treated with MG132 or bafilomycin as reported in **d,** and CLN3 levels were assessed by Real-Time PCR (N=two independent experiments, one-way Anova). **f,** Representative confocal images of CLN3 (red)-TGN46 (green)-LAMP1 (blue) triple staining on ARPE19 cells infected with pLVX-CLN3 and treated with 1µg/ml doxycycline for 4h, with and without a 12h doxycycline washout to visualize the mature protein. 50µg/ml CHX was added during the washout. CLN3-TGN46 (N=45 cells) and CLN3-LAMP1 (N=44 cells) Manders' co-localization coefficients are shown. One-way Anova. Scale bar 20µm, W/O, washout; Dox, doxycycline (****p<0.0001). Source data are provided as a Source Data file.

Supplementary Fig.3

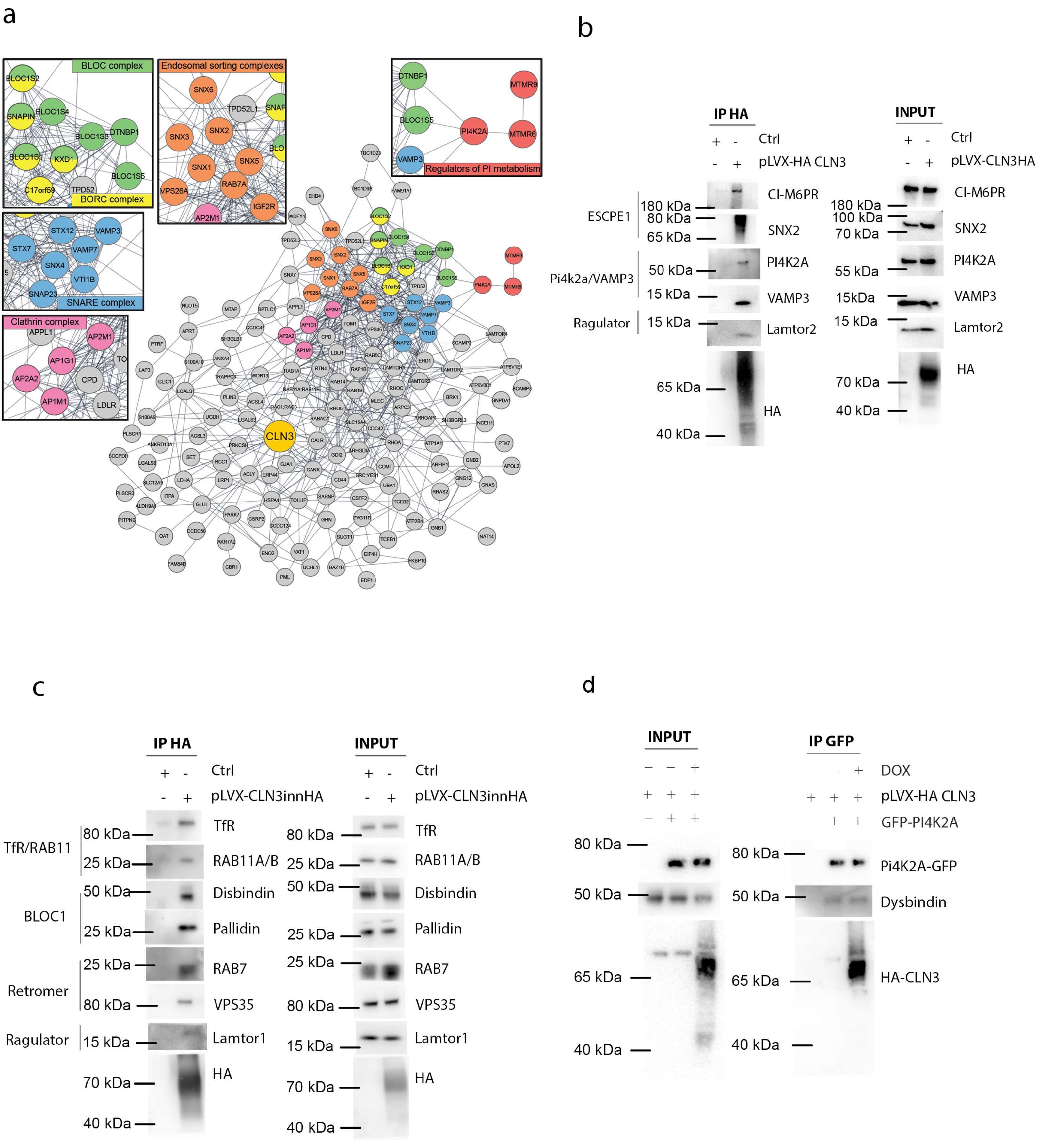

Supplementary Fig. 3 CLN3 interacts with endo-lysosomal trafficking and recycling complexes during starvation.

a, Interactome analysis in ARPE19-pLVX-CLN3*inn*HA cells induced for 24h with 1µg/ml doxycycline, and starved for 16h with serum and glutamine, immunoprecipitated for the HA-tag, showing enriched hits (fold change≥1.5, p-value<0.05) compared to cells not expressing the HA tag. Relevant protein complexes identified with Corum and displayed with Cytoscape, are color-coded and reported in separate enlargements. N=three independent experiments. Data are provided in Supplementary Data 1. P values are calculated using two-tailed unpaired t-tests. **b, c,** Cell lysates from HeLa cells with inducible expression of pLVX-CLN3*inn*HA or without HA expression, induced with 1µg/ml doxycycline for 40h, were incubated with HA beads and analyzed by immunoblotting to validate MS-interactome data. Repeated two times. **d,** GFP immunoprecipitation in HeLa-pLVX-CLN3*inn*HA stable cells ± doxycycline and transfected with PI4K2A-GFP or empty vector. Repeated two times.

Supplementary Fig.4

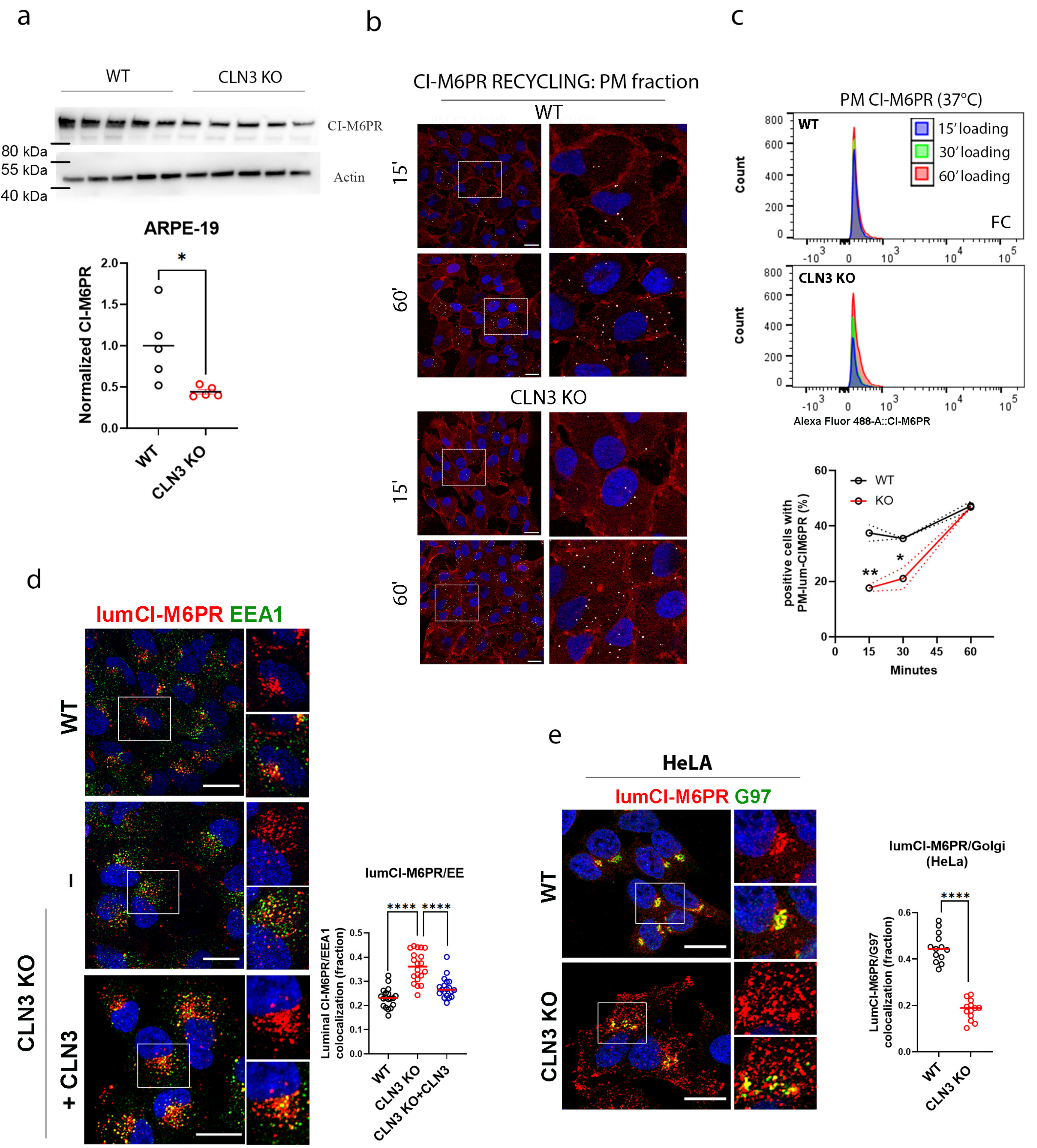

Supplementary Fig. 4 CI-M6PR recycling and trafficking are regulated by CLN3

a, Immunoblot analysis of CI-M6PR protein levels in WT and CLN3 KO ARPE19 cells (top, N=5 independent replicates), and its relative quantification (unpaired t test, two-tailed, *p<0.05). **b,** ARPE19 WT and CLN3-KO cells were loaded with 5µg/ml lumCI-M6PR at 4°C for 45 min, switched to 37°C for 15 or 60 min, and then stained for the plasma-membrane (PM) lumCI-M6PR fraction (white). Images were generated with the Imaris software. Scale bar 10µm. Crop shows enlargement areas. **c,** Representative flow cytometry histograms of ARPE19 WT and CLN3 KO cells, treated as in **b,** and chased at 37°C for 15, 30 or 60 min (repeated three times). The gating strategy is provided in Supplementary Fig. 9. Graphs show the percentage of positive cells at each time point normalized against the 15 min time point (two-way Anova, mean ± SEM showed with dotted lines). (*p<0.01, **p<0.01). **d,** ARPE19 WT, CLN3-KO and CLN3-KO+pLVX-CLN3-innHA cells (induced with doxycycline for 40h), were loaded with lumCI-M6PR antibody as reported in **4a** and stained for the indicated antibodies. Scale bar 20µm. Insets show enlargements areas. The quantification shows lumCI-M6PR-EEA1 Manders' colocalization coefficient (N=19-20 areas, mean+single values, three independent replicates, one-way Anova, ****p<0.0001). **e,** Representative confocal images of HeLa WT and CLN3-KO cells loaded with lumCI-M6PR (red) as in **d,** and co-stained with G97 (green). Insets show enlargements areas. Scale bar 20µm. The quantification shows lumCI-M6PR-G97 Manders' colocalization coefficient (N=19-20 areas, mean+single values, 3 independent replicates), unpaired t test (two-tailed) (****p<0.0001). Source data are provided as a Source Data file.

Supplementary Fig.5

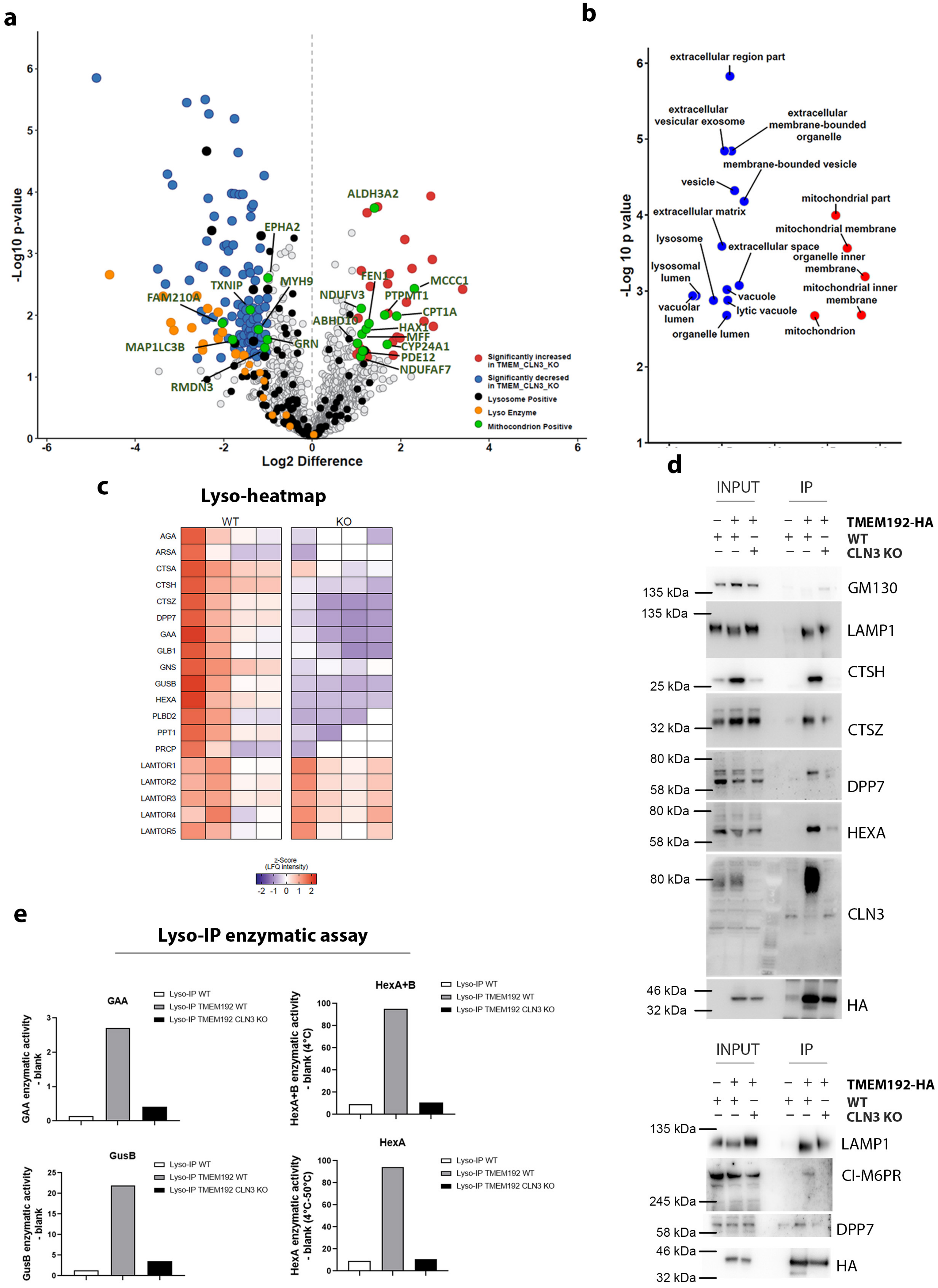

Supplementary Fig. 5 Reduction of lysosomal enzymes and accumulation of mitochondrial proteins in lysosomes of CLN3 KO cells

a, Volcano plots of the lysosomal proteome from ARPE19 WT and CLN3 KO cells from data shown in **5a**. Volcano plots highlight proteins significantly depleted (blue dots, fold change ≤-1, p-value<0.05) and enriched (red dots, fold change ≥1, p-value>0.05) compared to WT cells. Cells without HA expression were used as negative control. Mitochondrial proteins are reported in green.. N=4 independent experiments. Data are provided in Supplementary Data 2. P values are calculated using two-tailed unpaired t-tests. **b,** 1D annotation enrichment analysis of significantly reduced (blue) and increased (red) GOCC terms, relative to Lyso-IP proteomic data shown in Fig. **5a**. Data are provided in Supplementary Data 3. All the listed terms have a Benj. Hoch. FDR <0.05. **c,** Heatmaps of lysosomal genes relative to Lyso-IP MS proteomic data shown in Fig. **5a** and displaying the reduction of lysosomal enzymes in lysosomes from CLN3 KO cells. Proteins with decreased levels are shown in blue and with increased levels in red. **d,** Immunoblot analysis of lysosomal enzymes and CI-M6PR performed on the lysosomal fraction (Lyso-IP) of ARPE19 WT and CLN3 KO cells. Repeated two times. **e,** Enzymatic activities of GAA, GusB, HexA+B and HexA, measured on purified lysosomes (Lyso-IP samples) from ARPE19 WT and CLN3 KO. Source data are provided as a Source Data file.

Supplementary Fig.6

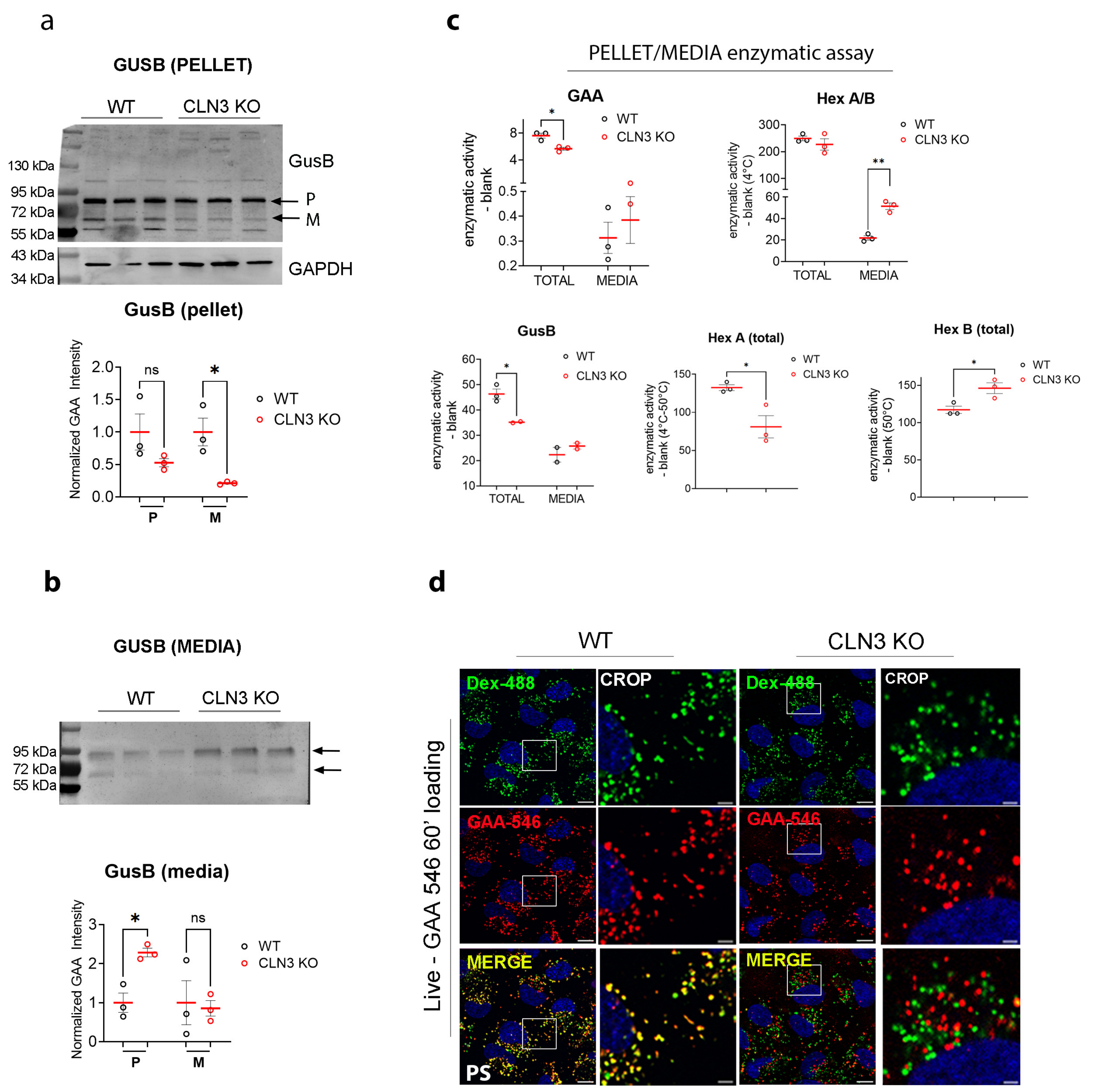

Supplementary Fig. 6 Lysosomal enzymes are secreted in the media in CLN3-KO cells

a, b, Immunoblot analysis of GusB protein maturation in the pellet (**a**) and media (**b**) of WT and CLN3 KO ARPE19 cells (N=3 independent replicates, Mean ± SEM, one-way Anova, *p<0.05). **c,** Enzymatic activities of GAA, GusB and HexA+B measured on cell lysates and supernatants from ARPE19 WT and CLN3 KO, and of HexA and HexB measured on cell lysates from ARPE19 WT and CLN3 KO. Mean ± SEM, unpaired t test (two-tailed) (*p<0.05, **p<0.01). **d,** representative live-image confocal snapshots of WT and CLN3-KO ARPE19 cells, loaded with 1µg/µl dextran for 1 hour the night before imaging, then washed for 16h in prolonged starvation media to label lysosomes (green), and loaded for 60 min with 40µg/ml rhGAA-546 before imaging. Scale bar 20µm. Enlargements areas are reported. Yellow staining shows the co-localization of rhGAA-546 and lysosomal dextran. Repeated two times. Source data are provided as a Source Data file.

Supplementary Fig.7

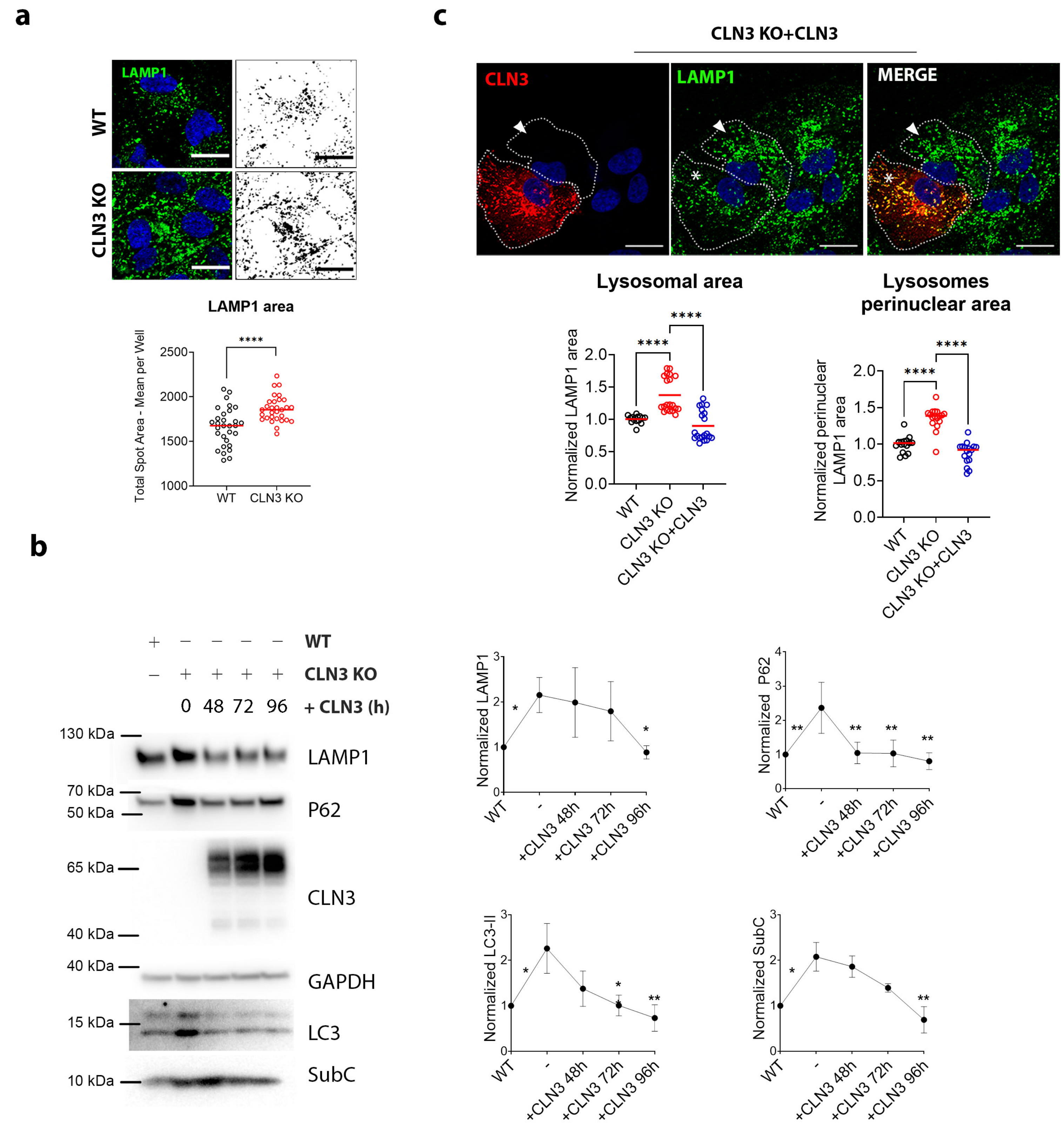

Supplementary Fig. 7 Lysosomal aggregation and impaired degradation in CLN3-KO cells
a, High-content analysis of LAMP1 staining in ARPE19 cells. Representative images and quantification of total lysosomal (LAMP1) area per cell. Results are mean+single values, unpaired t-test (two-tailed), three independent experiments, N=30 total fields per condition (****p<0.0001). **b,** Representative immunoblot of ARPE19 CLN3-KO+pLVX-CLN3innHA cells upon progressive doxycycline treatment (48, 72, 96h) to induce CLN3 reintroduction, and relative quantification. Mean ± SEM, three independent experiments, two-way Anova (*p<0.05, **p<0.01). **c,** Representative confocal images of ARPE19 WT, CLN3-KO and CLN3-KO+pLVX-CLN3 cells treated with 1µg/ml doxycycline for 40h, and stained for the indicated antibodies (arrow, aggregated lysosomes; asterisk, dispersed lysosomes, scale bar 20µm). Graphs show high-content quantification of lysosomal total and perinuclear area normalized against control cells (WT). Results are mean+single values, one-way Anova, three independent experiments, N=15-20 total fields per condition (****p<0.0001). Source data are provided as a Source Data file.

Supplementary Fig.8

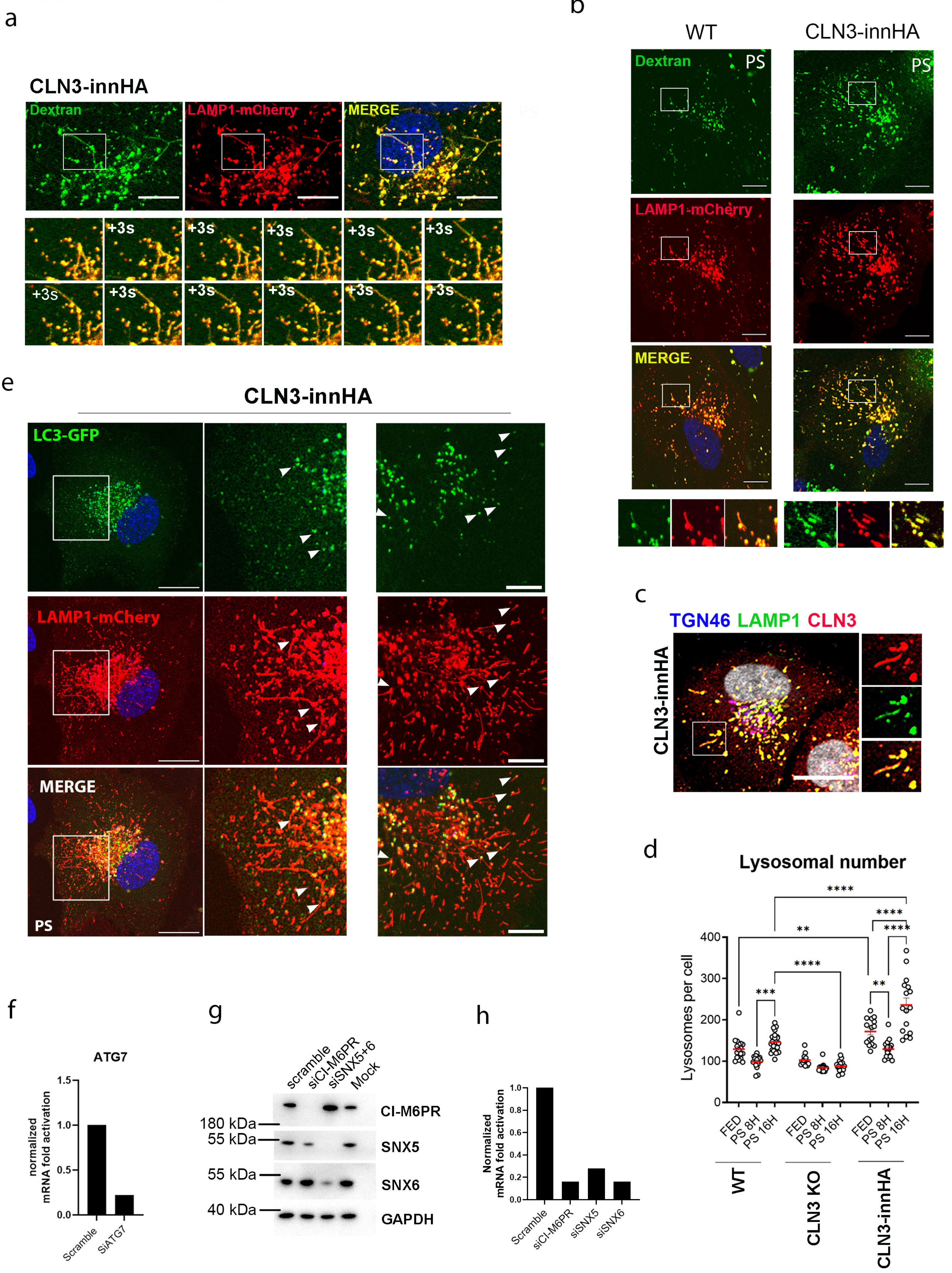

Supplementary Fig. 8 Lysosomal tubules are positive to dextran, LAMP1, and CLN3

a, Representative time-lapse images of CLN3*inn*HA cells, stably expressing LAMP1-mCherry and induced with 1µg/ml doxycycline for 40h, loaded with 1µg/µl Dextran for 1 hour the night before imaging, and then treated with PS media for 16h to label lysosomes (green). Scale bar 10µm. **b,** Representative live- images of ARPE19 WT and CLN3*inn*HA cells stably expressing LAMP1-mCherry and loaded with 1µg/µl dextran-488, treated as reported in Supplementary Fig. **8a**, showing lysosomal tubules positive to both markers. Scale bar 10µm. **c,** Representative confocal images of ARPE19 PLVX-CLN3*inn*HA cells+LAMP1mCherry, induced with 1µg/ml doxycycline for 40h and showing CLN3 and LAMP1 on tubules. Scale bar 20µm. **d,** Single values of data reported in Figure **7b** relative to lysosomes number per cell. Basal N=274-375 cells, 8h PS N=344-500 cells, 16h PS N=225-598 cells. Two-way Anova, mean + individual values (*p<0.05, **p<0.01, ***p<0.001, ****p<0.0001). **e,** pLVX-CLN3-innHA+LAMP1-mCherry cells were induced with doxycycline for 40h and transfected with LC3-GFP plasmid for 24h. Cells were then starved for 16h and analyzed by confocal microscopy. Repeated two times. **f,** ARPE19-pLVX-CLN3innHA cells were transfected with ATG7 or scramble siRNA for 72h and analyzed by Real-Time PCR. Scale bar 20µm (left), 10µm (right). N=three technical replicates. **g, h,** ARPE19 pLVX-CLN3innHA cells were transfected with scramble, CI-M6PR or SNX5/6 siRNA for 72h, and cells were analyzed by immunoblot (**g**) and real-time PCR (**h**) (N=three technical replicates). Source data are provided as a Source Data file.

Supplementary Fig.9

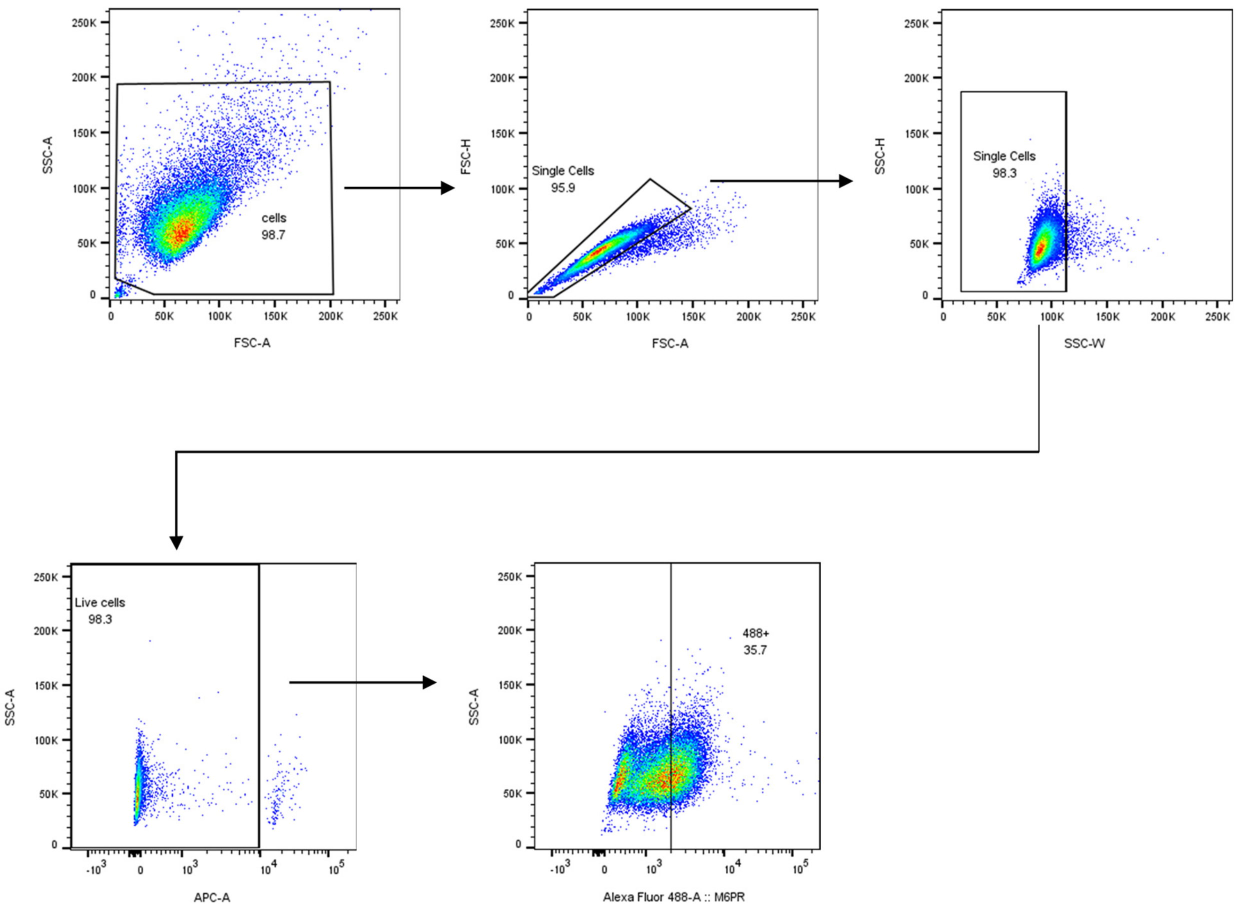

Supplementary Figure 9. Gating strategy for the flow cytometric analyses.

Single cells were selected using a conservative three-step process as follows: (a) forward scatter-area vs side scatter-area, (b) forward scatter-area vs forward scatter-height, and (c) side scatter-width vs side scatter-height dot plots. (d) Live single cells were then identified from viability (Helix non-permeant NIR, Biolegend in the APC channel) vs side scatter-area dot plot. (e) Finally, AB +ve cells were gated for quantification.
