# Supplementary material for: Loss of the batten disease protein CLN3 leads to mis-trafficking of M6PR and defective autophagic-lysosomal reformation
Source: Nat Commun. 2023 Jul 3;14:3911. doi: 10.1038/s41467-023-39643-7 (PMC10317969; doi:10.1038/s41467-023-39643-7)
Supplement: Supplementary file 3 — Description of Additional Supplementary Files [file 41467_2023_39643_MOESM3_ESM.pdf]

File name: Supplementary Data 1
Description: Complete list of CLN3 interactors in basal and starvation conditions. P values are calculated using two-tailed unpaired t-tests.

File name: Supplementary Data 2
Description: Proteome analysis of lysosomal content relative to ARPE19 WT+TMEM192-HA and CLN3-KO+TMEM192-HA cells. Cells with no TMEM192-HA expression are used as negative control of the lysosomal immunopurification protocol. P values are calculated using two-tailed unpaired t-tests.

File name: Supplementary Data 3
Description: 1D annotation enrichment analysis of GOCC, relative to Lyso-IP proteomic data. All the listed terms have a Benj. Hoch. FDR <0.05.

File name: Supplementary Movie 1
Description: Live-imaging Airyscan (AS) confocal super resolution microscopy of ARPE19 pLVX-CLN3innHA cells induced with doxycycline for 40h and starved with serum+glutamine for 16h. Lysosomal tubules are labelled with LAMP1-mCherry (5 frames/sec).

File name: Supplementary Movie 2
Description: Confocal super resolution microscopy of ARPE19 WT cells starved with serum+glutamine for 16h. Lysosomal tubules are labelled with dextran (5 frames/sec).

File name: Supplementary Movie 3
Description: Confocal super resolution microscopy of ARPE19 CLN3 KO cells starved with serum+glutamine for 16h. Lysosomal tubules are labelled with dextran (5 frames/sec).
