# Supplementary material for: Loss of the batten disease protein CLN3 leads to mis-trafficking of M6PR and defective autophagic-lysosomal reformation
Source: Nat Commun. 2023 Jul 3;14:3911. doi: 10.1038/s41467-023-39643-7 (PMC10317969; doi:10.1038/s41467-023-39643-7)
Supplement: Supplementary file 10 — Reporting Summary [file 41467_2023_39643_MOESM10_ESM.pdf]

nature portfolio

Corresponding author(s): Andrea Ballabio, Alessia Calcagni

Last updated by author(s): Jun 13, 2023

Reporting Summary

Nature Portfolio wishes to improve the reproducibility of the work that we publish. This form provides structure for consistency and transparency in reporting. For further information on Nature Portfolio policies, see our Editorial Policies and the Editorial Policy Checklist.

Statistics

For all statistical analyses, confirm that the following items are present in the figure legend, table legend, main text, or Methods section.

n/a	Confirmed	
☐	☒	The exact sample size (*n*) for each experimental group/condition, given as a discrete number and unit of measurement
☐	☒	A statement on whether measurements were taken from distinct samples or whether the same sample was measured repeatedly
☐	☒	The statistical test(s) used AND whether they are one- or two-sided<br>*Only common tests should be described solely by name; describe more complex techniques in the Methods section.*
☒	☐	A description of all covariates tested
☐	☒	A description of any assumptions or corrections, such as tests of normality and adjustment for multiple comparisons
☐	☒	A full description of the statistical parameters including central tendency (e.g. means) or other basic estimates (e.g. regression coefficient) AND variation (e.g. standard deviation) or associated estimates of uncertainty (e.g. confidence intervals)
☐	☒	For null hypothesis testing, the test statistic (e.g. *F*, *t*, *r*) with confidence intervals, effect sizes, degrees of freedom and *P* value noted<br>*Give P values as exact values whenever suitable.*
☒	☐	For Bayesian analysis, information on the choice of priors and Markov chain Monte Carlo settings
☒	☐	For hierarchical and complex designs, identification of the appropriate level for tests and full reporting of outcomes
☒	☐	Estimates of effect sizes (e.g. Cohen's *d*, Pearson's *r*), indicating how they were calculated

Our web collection on statistics for biologists contains articles on many of the points above.

Software and code

Policy information about availability of computer code

Data collection	Zeiss Zen Blue software, FACSDiva software
Data analysis	Graphpad Prism 8; Imaris 9.5.1; Fiji 1.0; Microsoft Excel 2016; Columbus 2.6.0, Perkin Elmer;FlowJo v10; MaxQuant (1.6.2.10); Corum; Cytoscape 3.10.0; Perseus 1.6.2.3.

For manuscripts utilizing custom algorithms or software that are central to the research but not yet described in published literature, software must be made available to editors and reviewers. We strongly encourage code deposition in a community repository (e.g. GitHub). See the Nature Portfolio guidelines for submitting code & software for further information.

Data

Policy information about availability of data

All manuscripts must include a data availability statement. This statement should provide the following information, where applicable:

- Accession codes, unique identifiers, or web links for publicly available datasets
- A description of any restrictions on data availability
- For clinical datasets or third party data, please ensure that the statement adheres to our policy

Full scans for all western blots as well as source data for all the graphs are provided with this manuscript. For graphs, the exact p value for all the experiments is present in the Source data file. All other data are available from the corresponding author on reasonable request. The MS proteomics data were deposited to the

ProteomeXchange Consortium through the PRIDE partner repository with the dataset identifier PXD031582. To identify enriched GO terms in the Lyso-IP dataset, we utilized the 1D enrichment tool in Perseus. The protein-protein interaction network was built in the Cytoscape environment.

Research involving human participants, their data, or biological material

Policy information about studies with human participants or human data. See also policy information about sex, gender (identity/presentation), and sexual orientation and race, ethnicity and racism.

Reporting on sex and gender	N/A
Reporting on race, ethnicity, or other socially relevant groupings	N/A
Population characteristics	N/A
Recruitment	N/A
Ethics oversight	N/A

Note that full information on the approval of the study protocol must also be provided in the manuscript.

Field-specific reporting

Please select the one below that is the best fit for your research. If you are not sure, read the appropriate sections before making your selection.

☒ Life sciences ☐ Behavioural & social sciences ☐ Ecological, evolutionary & environmental sciences

For a reference copy of the document with all sections, see nature.com/documents/nr-reporting-summary-flat.pdf

Life sciences study design

All studies must disclose on these points even when the disclosure is negative.

Sample size	The sample size was chosen based on previous experience with respect to how many number of cells per treatment group are required to reliably detect biologically meaningful differences among groups. Additionally, experiments were repeated at least three times (unless differently stated) and sample size was further defined accordingly to the consistency and significance of measured differences between groups and conditions. For high content imaging, thousands of cells/group were analyzed. The exact sample size for each experiment is reported in each figure legend, and in the "Statistics and Reproducibiliy" statement in the Methods section.
Data exclusions	We did not apply any exclusion criteria
Replication	All experiments were carried out under standard and clearly defined conditions, and were replicated successfully by at least one researcher. All attempts at replication were successful. Experiments were repeated at least three times (unless differently stated).This information is reported in each figure legend, and in the "Statistics and Reproducibiliy" statement in the Methods section.
Randomization	The cells used in the manuscript were randomly assigned to the experimental group/treatment. Also, images were selected randomly and analyzed equally, no sub-sampling was performed, so no randomization was necessary.
Blinding	Investigators were blind during immunofluorescence analyses and image acquisition. For WB experiments, blinding is not a standard in the field, however WB experiments were double checked by at least two researchers.

Reporting for specific materials, systems and methods

We require information from authors about some types of materials, experimental systems and methods used in many studies. Here, indicate whether each material, system or method listed is relevant to your study. If you are not sure if a list item applies to your research, read the appropriate section before selecting a response.

Materials & experimental systems

n/a	Involved in the study
☐	☒ Antibodies
☐	☒ Eukaryotic cell lines
☒	☐ Palaeontology and archaeology
☒	☐ Animals and other organisms
☒	☐ Clinical data
☒	☐ Dual use research of concern
☒	☐ Plants

Methods

n/a	Involved in the study
☒	☐ ChIP-seq
☐	☒ Flow cytometry
☒	☐ MRI-based neuroimaging

Antibodies

Antibodies used

The polyclonal anti-CLN3 antibody is a home-made antibody, obtained by immunizing rabbits with a purified Escherichia Coli expressed His-tagged CLN3 polypeptide, containing both luminal and cytoplasmic protein domains. The his-tagged CLN3 polypeptide was generated in-house while injection in rabbits was performed by Covalab.

The following commercial antibodies were used in this study: HA (Biolegend, Cat# 902301; Cat# 901501, 1:400 dilution for IF, 1:1000 for WB), LAMP1 (Santa Cruz, Cat# sc-20011, 1:200 dilution for IF, 1:1000 dilution for WB), GM130 (abcam, ab52649, 1:400 dilution for IF), TGN46 (Bio-Rad, AHP500GT, 1:400 dilution for IF), LAMP1 (Hybridoma Bank, Cat# H4A3-a, 1:500 dilution for immunoEM), LC3 (Novus, Cat#NB100-2220 1:1000 dilution for WB), p62 (Novus 2C11 Cat# H00008878-M01, 1:1000 dilution for WB), NBR1 (Abnova, cat# H00004077-M01, 1:1000 dilution for WB), ATP synthase C (Abcam, Cat# ab181243, 1:1000 dilution for WB), PI4K2A (Santa Cruz, Cat# sc-390026, 1:1000 dilution for WB), Pallidin (Cat# 10891-2-AP, Proteintech, 1:500 dilution for WB), Dysbindin (Novus, Cat# NBP2-16245, 1:1000 dilution for WB), Rab7 (CellSignaling, Cat# 9367S, 1:1000 dilution for WB), VPS35 (Abcam, ab10099, 1:1000 dilution for WB), Lamtor1 (CellSignaling, Cat# 8975, 1:1000 dilution for WB), Lamtor2 (CellSignaling, Cat# 8145, 1:1000 dilution for WB), VAMP3 (Novus, NB300-510, 1:1000 dilution for WB), TfR (Thermo, Cat# 13-6890 1:1000 dilution for WB), Rab11 (Prointech, Cat# 15903-1-AP, 1:1000 dilution for WB), CI-M6PR (Abcam, Cat# ab32815, 1:1000 dilution for WB and Novus, 2G11 Cat# NB300-514SS, 1:200 dilution for IF), Cathepsin H (Santa Cruz, Cat# sc-398527, 1:1000 dilution for WB), Cathepsin X/Z/P (R&D, Cat# AF934, 1:1000 dilution for WB), Cathepsin D (abcam, Cat# ab75852, 1:1000 dilution for WB), DPP7 (Novus Biologicals, Cat# NBP132875, 1:1000 dilution for WB), HexA (Abcam, ab189865, 1:1000 dilution for WB), GAPDH (Santa Cruz, sc-32233 1:5000 dilution for WB). HRP-conjugated secondary antibodies to Mouse (Cat# 401215 - 1:6000 dilution) and Rabbit (Cat# 401315 - 1:6000 dilution). HA–Agarose (Sigma, Cat# A2095), HA-magnetic beads (Thermo, Cat# 88836), GFP (Cromotek, Cat# GTA-20), anti-rabbit IgG (Bethyl, Cat.# P120-101), ProteinA-Sepharose 4B Conjugate (Invitrogen, Cat#101041). Donkey anti-Rabbit IgG (H+L) Alexa Fluor 488 (Cat# A-21206 - 1:500 dilution), Alexa Fluor 568 (Cat# A-10042 -1:500 dilution), Donkey anti-mouse IgG (H+L) Alexa Fluor 568 (Cat# A-10037 - 1:500 dilution), Alexa Fluor 647 (Cat# A-31571 - 1:500 dilution), Alexa Fluor 594 (Cat# A-21203 - 1:500 dilution), Donkey anti-goat IgG (H+L) Alexa Fluor 647 (Cat# A-21447 - 1:500 dilution), Donkey anti-sheep (H+L) Alexa Fluor 488 (Cat# A-11015 - 1:500 dilution) were from Thermo Fisher Scientific.

Validation

The home-made anti-CLN3 antibody has been validated in human cells, by using CLN3-depleted and CLN3-overexpressing cell lines through multiple approaches. In Fig.1 we validated the antibody through IF staining in wt and CLN3-KO cells, Airyscan super-resolution microscopy, immunoprecipitation of the endogenous protein, and immunoblot detection of CLN3 in a doxycyclin-inducible CLN3 overexpression line after different stimulation protocols with the inducer. In Supplementary Fig.1 we validated the antibody in WT and CLN3-KO cell lines through IF and WB. In Supplementary Fig.2 we validated the antibody in WT and CLN3-KO cell lines upon lysosomal-immunopurification, treatments with proteasome and autophagy inhibitors, and we assessed CLN3 degradation in dox-inducible CLN3-overexpressing lines upon CHX+dox removal treatments, and CLN3 localization during different dox-stimulation protocols.

Most of the antibodies used in the study were bought from commercial vendors and were validated by the manufacturers and/or other studies. Some of the antibodies were further validated using KO/knocked-down cell lines.
See individual antibody's web page (link shown below) on the manufacture's website for validation and relevant citations:
HA (rabbit): https://www.biolegend.com/en-us/products/purified-anti-ha-11-epitope-tag-antibody-11375
HA (mouse): https://www.biolegend.com/en-us/products/purified-anti-ha-11-epitope-tag-antibody-11374
LAMP1: https://www.scbt.com/it/p/lamp-1-antibody-h4a3
GM130: https://www.abcam.com/products/primary-antibodies/gm130-antibody-ep892y-cis-golgi-marker-ab52649.html
TGN46: https://www.bio-rad-antibodies.com/polyclonal/human-tgn46-antibody-ahp500.html?f=purified
LAMP1 (Hybridoma bank): https://dshb.biology.uiowa.edu/H4A3
Ricciardi, Simona et al. “The role of NSP6 in the biogenesis of the SARS-CoV-2 replication organelle.” Nature vol. 606,7915 (2022): 761-768. doi:10.1038/s41586-022-04835-6
LC3: https://www.novusbio.com/products/lc3b-antibody_nb100-2220
p62: https://www.novusbio.com/products/p62-sqstm1-antibody-2c11_h00008878-m01
NBR1: https://www.abnova.com/products/products_detail.asp?catalog_id=H00004077-M01
Recombinant Anti-ATP synthase C antibody: https://www.abcam.com/products/primary-antibodies/atp-synthase-c-antibody-epr13907-ab181243.html
PI4K2A: https://www.scbt.com/p/pi-4-kinase-ii-alpha-antibody-b-5?requestFrom=search
PLDN: https://www.ptglab.com/products/PLDN-Antibody-10891-2-AP.htm
Dysbindin: https://www.novusbio.com/products/dysbindin-antibody_nbp2-16245
RAB7: https://www.cellsignal.com/products/primary-antibodies/rab7-d95f2-xp-rabbit-mab/9367
VPS35: https://www.abcam.com/products/primary-antibodies/vps35-antibody-ab10099.html
Lamtor1: https://www.cellsignal.com/products/primary-antibodies/lamtor1-c11orf59-d11h6-xp-rabbit-mab/8975
Lamtor2: https://www.cellsignal.com/products/primary-antibodies/lamtor2-robld3-d7c10-rabbit-mab/8145

VAMP3: https://www.novusbio.com/products/vamp3-cellubrevin-antibody_nb300-510
TfR: https://www.thermofisher.com/antibody/product/Transferrin-Receptor-Antibody-clone-H68-4-Monoclonal/13-6890
Rab11: https://www.ptglab.com/products/RAB11A-Antibody-15903-1-AP.htm
CI-M6PR: https://www.abcam.com/products/primary-antibodies/m6pr-cation-independent-antibody-ab32815.html
CI-M6PR: https://www.novusbio.com/products/igf-ii-r-igf2r-antibody-2g11_nb300-514
Cathepsin H: https://www.scbt.com/p/cathepsin-h-antibody-f-7?requestFrom=search
Cathepsin X/Z/P: https://www.rndsystems.com/products/human-mouse-rat-cathepsin-x-z-p-antibody_af934
Cathepsin D: https://www.abcam.com/products/primary-antibodies/cathepsin-d-antibody-epr3057y-ab75852.html
DPP7: https://www.novusbio.com/search?keywords=NBP132875
HEXA: https://www.abcam.com/products/primary-antibodies/hexa-antibody-c-terminal-ab189865.html
GAPDH: https://www.scbt.com/p/gapdh-antibody-6c5?requestFrom=search

Eukaryotic cell lines

Policy information about cell lines and Sex and Gender in Research

Cell line source(s): The cell lines used in this study were ARPE19, Hela and HEK293T cells. Cells were purchased from ATCC. ARPE19 and HeLa CLN3-KO and inducible CLN3-overexpressing cells were generated in this study.

Authentication: ARPE19 and HeLa CLN3-KO cells were authenticated by WB, RT-PCR or sequencing.
Silencing of target genes was validated by WB or RT-PCR. Commercial cell lines (ARPE19, HeLa and HEK293) were purchased recently from ATCC and validated by morphological analysis

Mycoplasma contamination: Cells were routinely tested and validated for the absence of mycoplasma

Commonly misidentified lines (See ICLAC register): No commonly misidentified lines were used.

Flow Cytometry

Plots

Confirm that:

☒ The axis labels state the marker and fluorochrome used (e.g. CD4-FITC).

☒ The axis scales are clearly visible. Include numbers along axes only for bottom left plot of group (a 'group' is an analysis of identical markers).

☒ All plots are contour plots with outliers or pseudocolor plots.

☒ A numerical value for number of cells or percentage (with statistics) is provided.

Methodology

Sample preparation: Cells were cultured in the following media: HeLa and HEK 293T in DMEM (Cat# 16777-200, VWR), ARPE19 in DMEM-F12 (Cat# 11320082, Thermo Fisher Scientific) and DMEM/F-12, no glutamine (Cat# 21331-020, Gibco). All media were supplemented with 10% inactivated FBS (tetracycline-free) (Cat# 631106, Clontech/Takara), 2 mM glutamine (Cat# 25030081, ThermoFisher), penicillin (100 IU/mL) and streptomycin (100 μg/mL) (Cat# 10378016, ThermoFisher) and maintained at 37°C and 5% CO2. Stable Tet-On ARPE19 CLN3-innHA, CLN3 and HeLa CLN3-innHA cell lines were generated by transducing cells with the lenti-pLVX Tet-One Inducible Expression System (Clontech). Cells were processed either for biochemical, FC, immuno-staining or live-imaging analyses as reported in the methods section. For PM-CI-M6PR FC experiments, cells were quickly washed with media+BSA1%, and then incubated at 4°C for 45 min with a pre-cooled mix containing saturating concentrations of the monoclonal anti-CI-M6PR (Cat# NB300-514, Clone 2G11, Novus) antibody in complete media+10 mM HEPES. For recycled PM CI-M6PR, cells were incubated with the antibody at 37°C for 15, 30 or 60 min in complete media. After primary antibody incubation, cells were washed once with 2ml of ice-cold FC buffer (PBS, 1% FBS, 2mM EDTA), centrifuged at 300g for 5 min, and then incubated with a pre-cooled mix containing the specific secondary antibody (1:500), for 45 min at 4°C. Cells were then washed once, and incubated with the Helix NP™ NIR (Biolegend, cat# 425301) dye, a far-red emitting nucleic acid stain used for the discrimination of live and dead cells, and analyzed.

Instrument: Flow cytometry data were acquired on a Becton Dickinson and Company (BD) Fortessa Analyzer equipped with 405, 488, 640nm lasers and FACSDiva software after performance testing using CS&T beads (BD).
Microscopy data were generated with an LSM 710 or LSM 880 + Airyscan systems (Carl Zeiss), equipped with a full incubation chamber. For enzymatic assays, samples were analyzed on a Promega GloMax Multidetection system fluorimeter. MS spectrometry data were generated with a NanoLC 1200 coupled via a nano-electrospray ionization source to the quadrupole-based Q Exactive HF benchtop mass spectrometer. For EM experiments, samples were sectioned using a Leica EM UC7 ultramicrotome (Leica Microsystems, Vienna, Austria). EM images were acquired from thin sections using a FEI Tecnai-12 electron microscope (FEI, Eindhoven, Netherlands) equipped with a VELETTA CCD digital camera (Soft Imaging Systems GmbH, Munster, Germany).

Software: The FACSDiva software was used to collect the data, while FlowJo (v10.8.1) was used for data analysis.

Cell population abundance: Blood bank saline solution (Thermo Scientific) was used as the sheath fluid. Single cells were identified by excluding for debris and cell aggregates via serial plots of side scatter (ssc)-a/ forward scatter (fsc)-a, fsc-h/fsc-a, and ssc-h and ssc-w. Spectral compensation was set based on single-color controls. Viability was determined using Helix NIR (Biolegend) under 640nm

excitation and acquired in the APC channel through a 670/14 band-pass filter. FITC was excited at 488nm and acquired in the FITC channel through 530/30 BP and 505 long-pass filters. At least 10,000 live cells were acquired for each experimental sample.

Gating strategy

Positive gates were set by first accounting for autofluorescence in the unstained sample, positive signal in the single-stained sample, then any additional signal from a secondary-antibody only sample.

☒ Tick this box to confirm that a figure exemplifying the gating strategy is provided in the Supplementary Information.
