# Supplementary material for: Loss of the batten disease protein CLN3 leads to mis-trafficking of M6PR and defective autophagic-lysosomal reformation
Source: Nat Commun. 2023 Jul 3;14:3911. doi: 10.1038/s41467-023-39643-7 (PMC10317969; doi:10.1038/s41467-023-39643-7)

Figure 1d

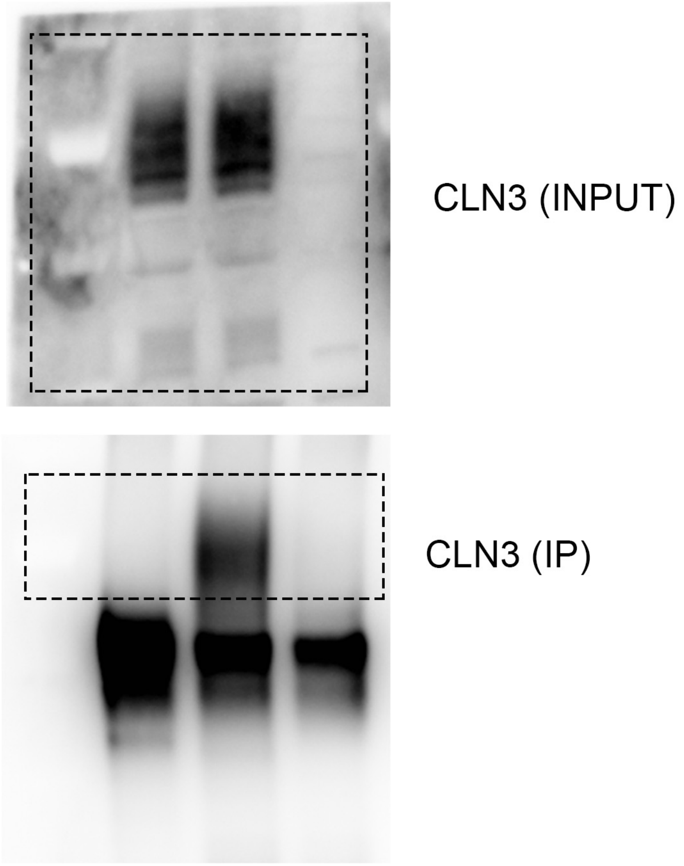

Figure 1e

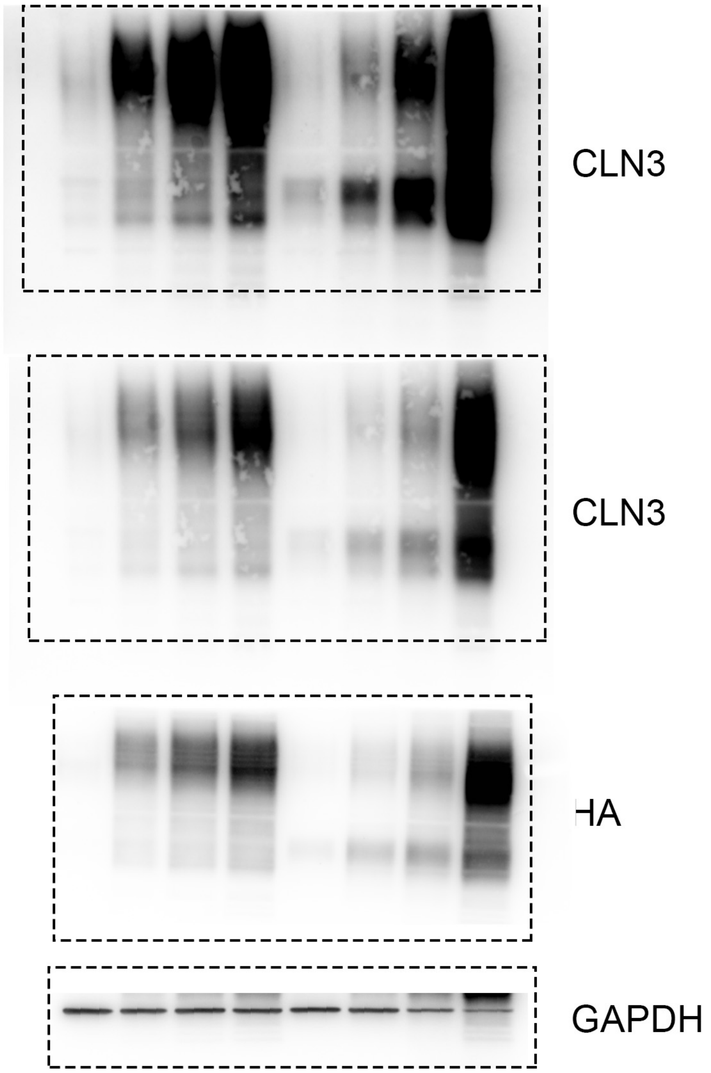

Figure 3a

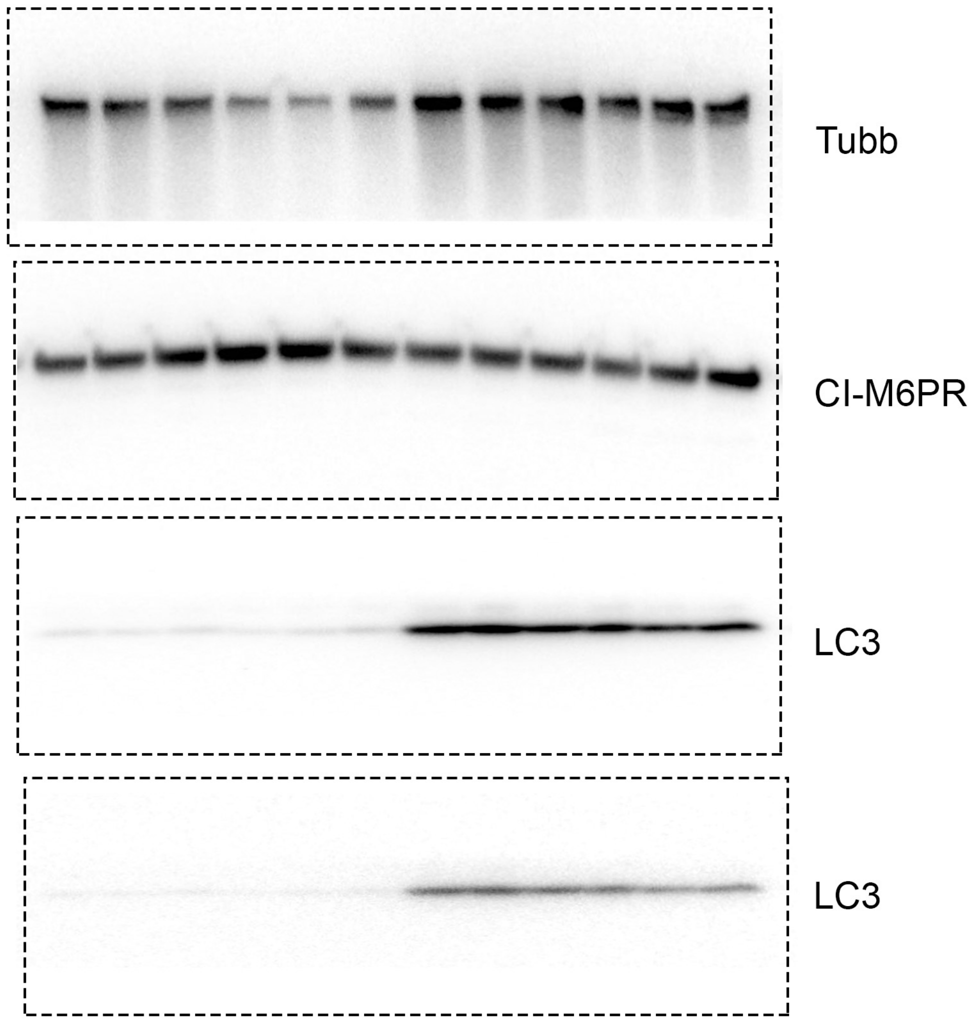

Figure 3d

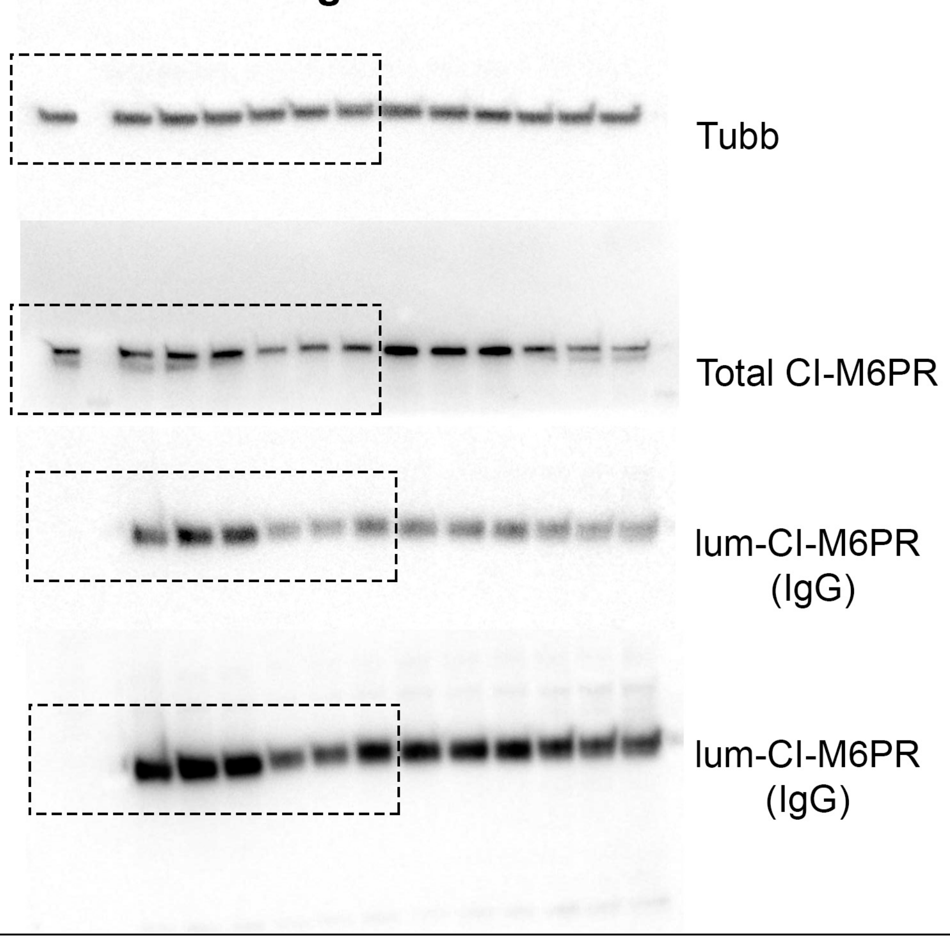

Figure 5b

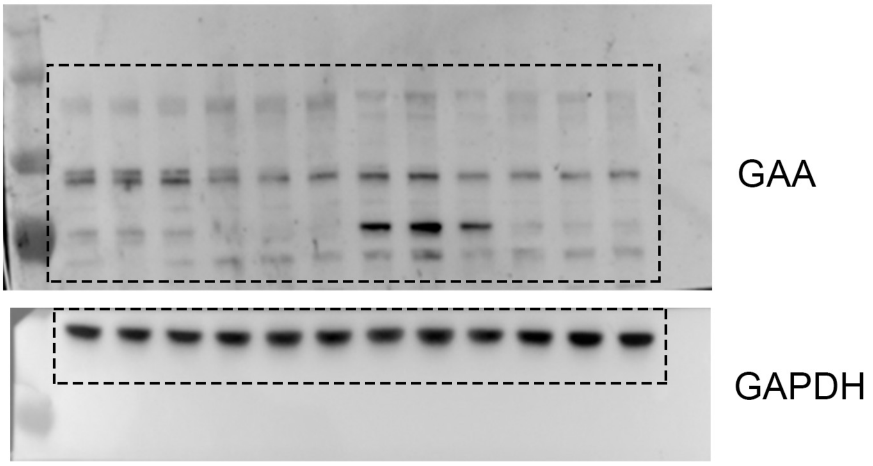

Figure 5c

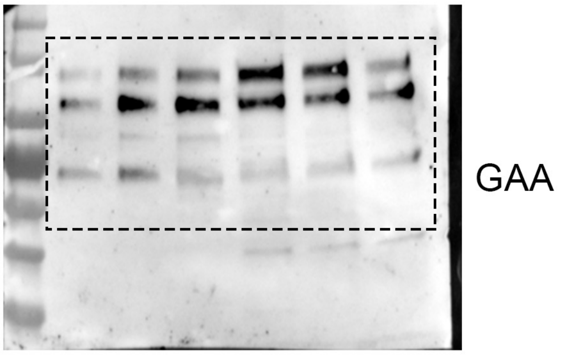

Figure 6c

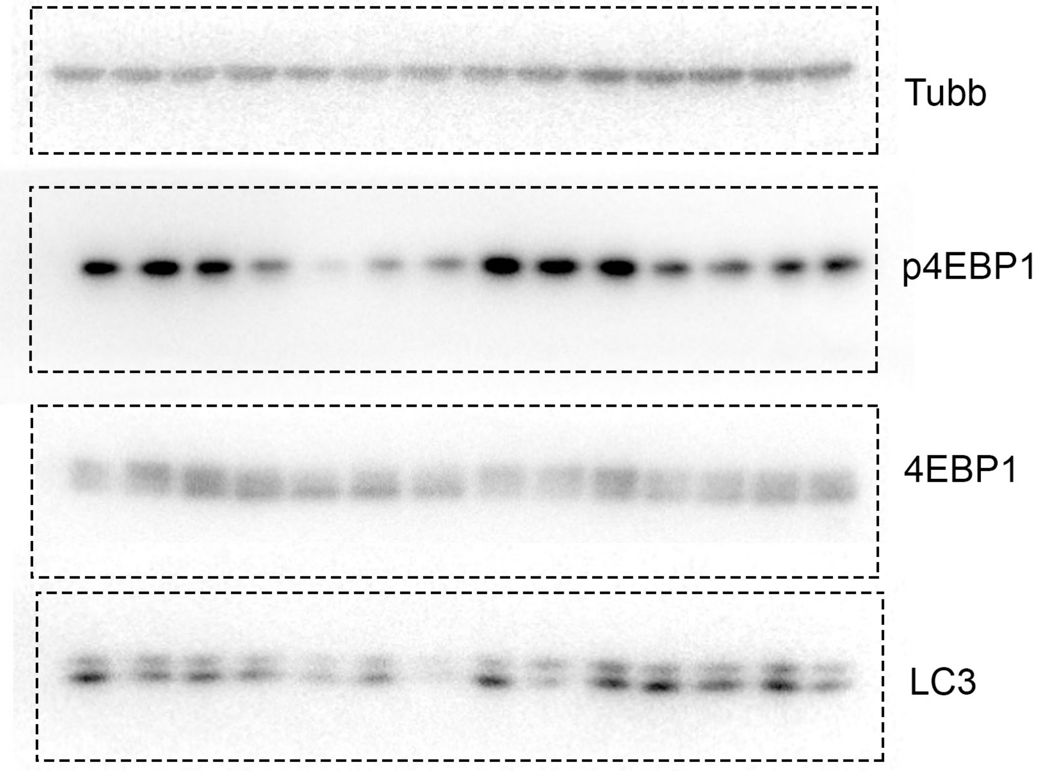

Figure 8a

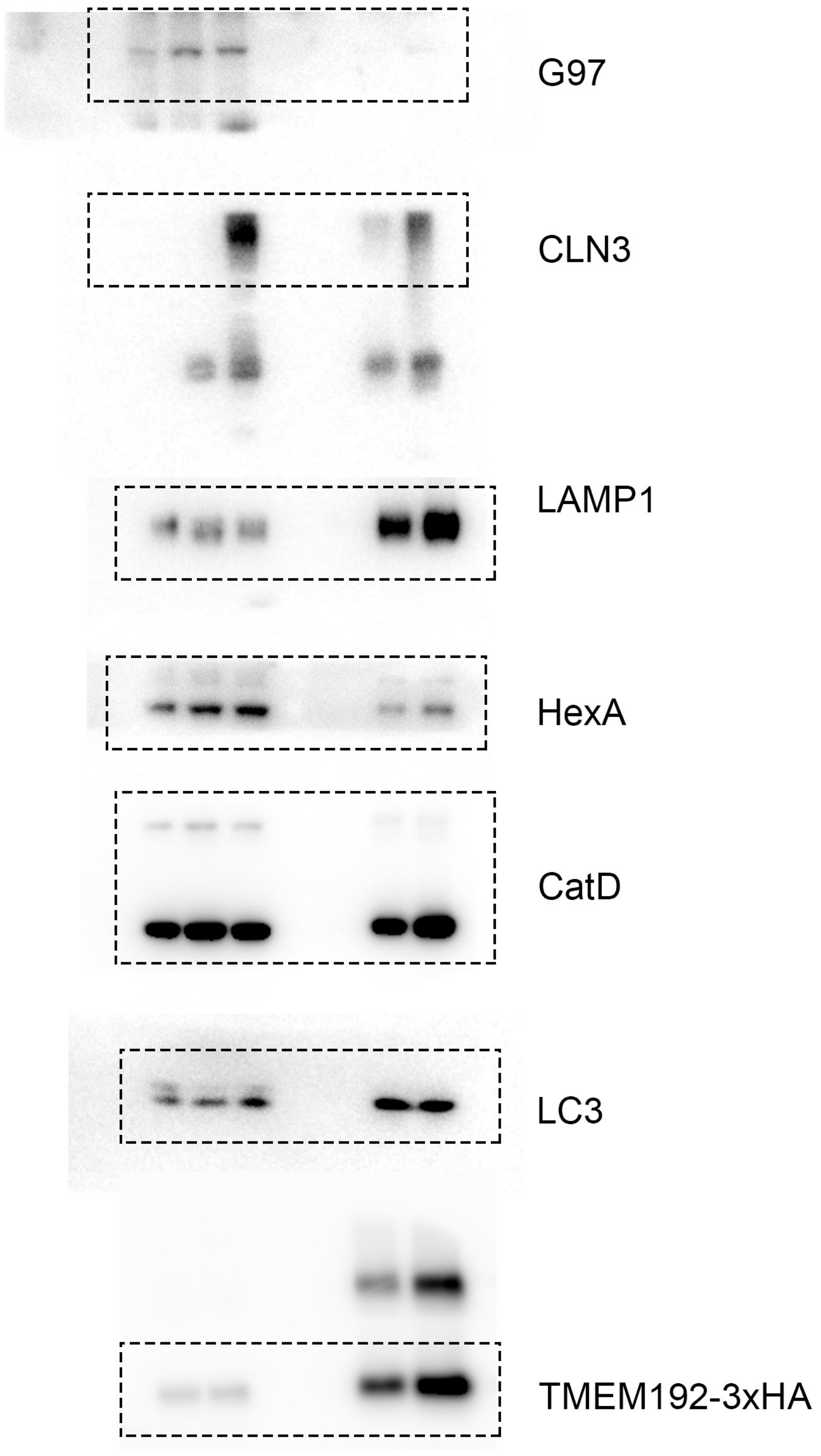

Supplementary Fig.1f

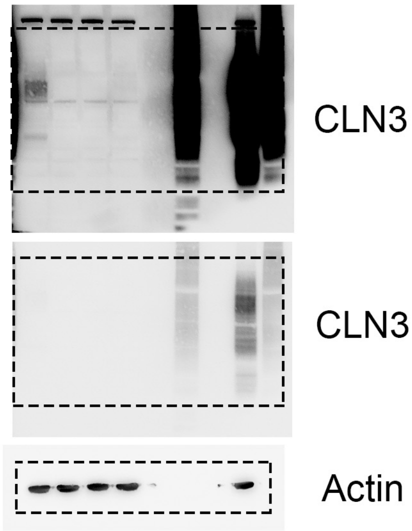

Supplementary Fig.1h

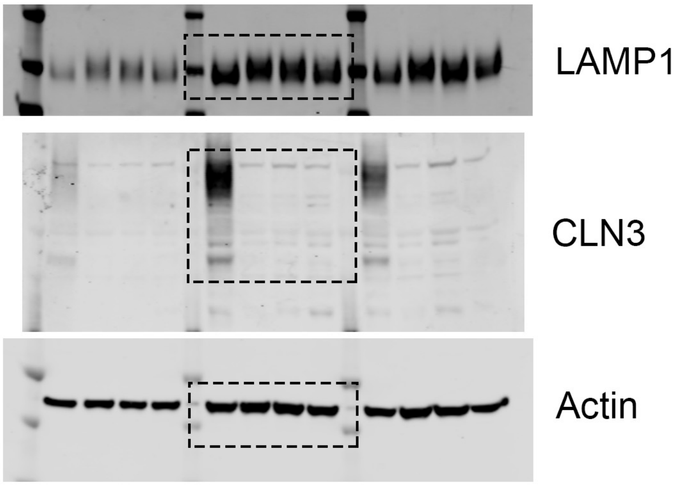

Supplementary Fig.2a

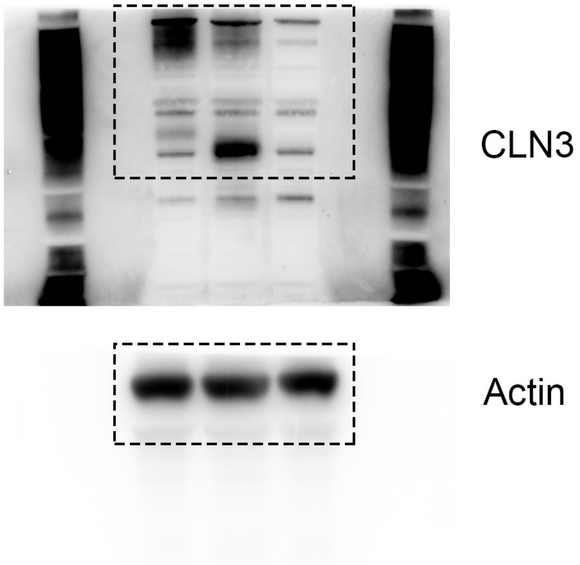

Supplementary Fig.2c

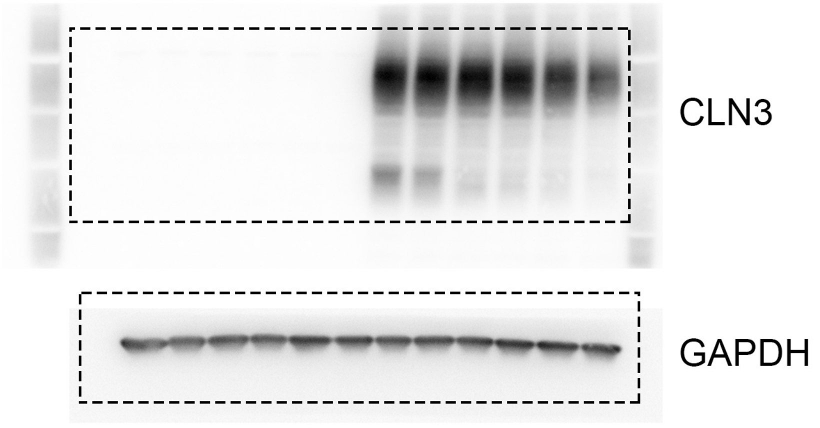

Supplementary Fig.2b

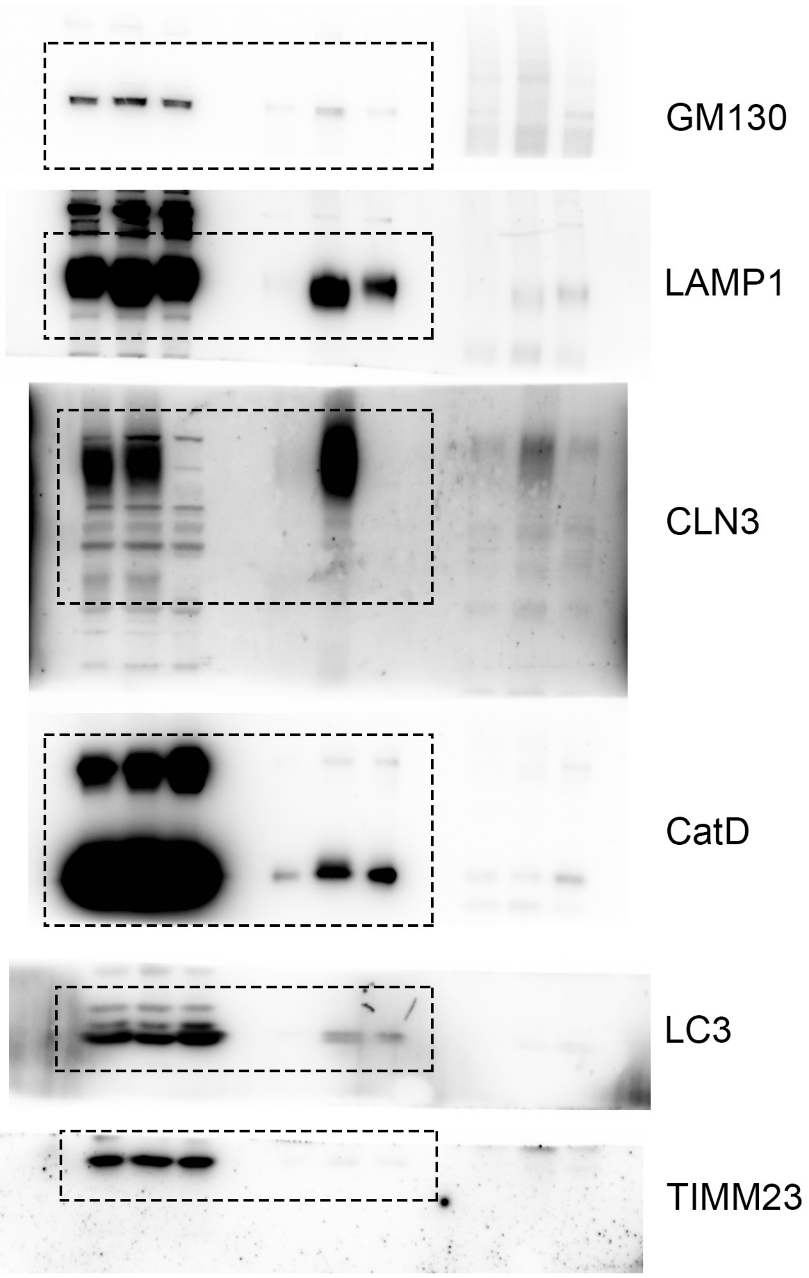

Supplementary Fig.2d

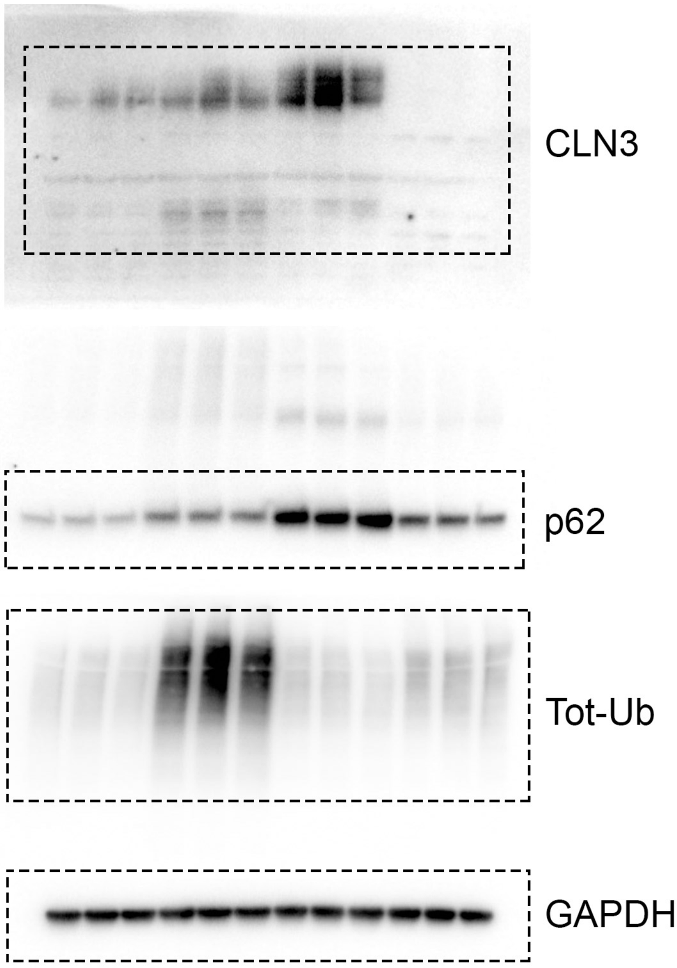

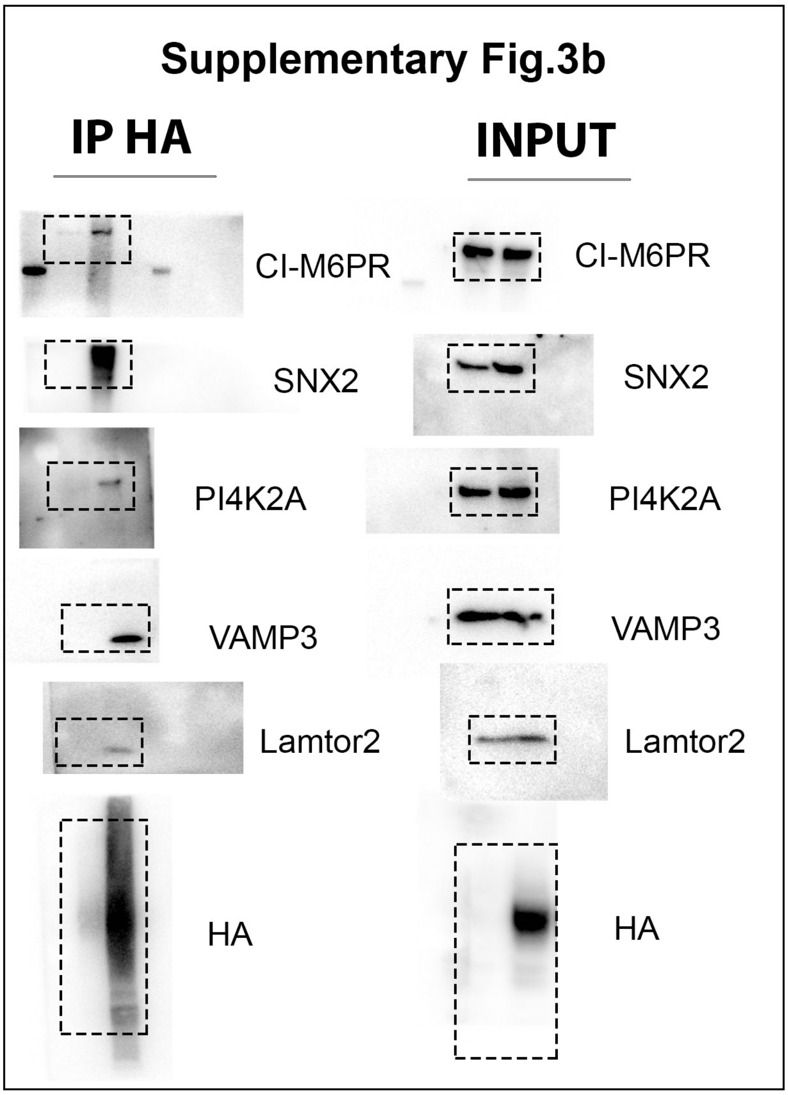
Supplementary Fig.3b
IP HA
INPUT
CI-M6PR
SNX2
PI4K2A
VAMP3
Lamtor2
HA
CI-M6PR
SNX2
PI4K2A
VAMP3
Lamtor2
HA

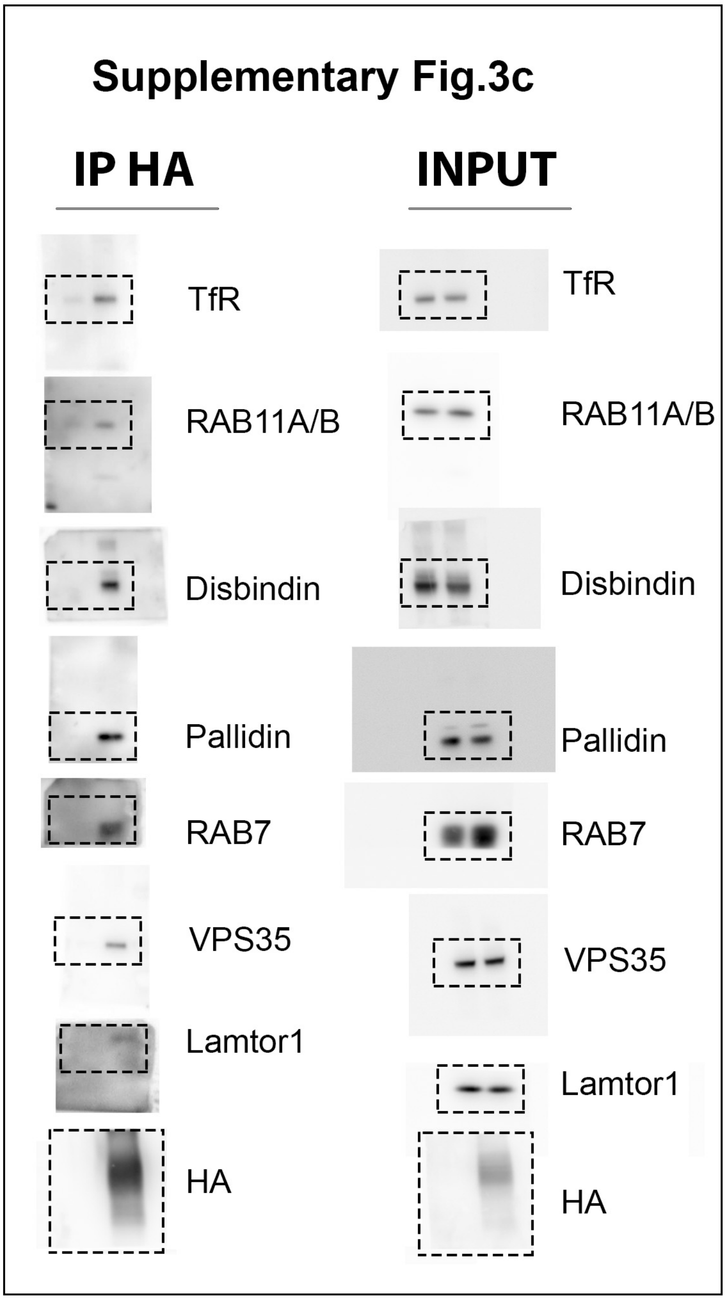
Supplementary Fig.3c
IP HA
INPUT
TfR
RAB11A/B
Disbindin
Pallidin
RAB7
VPS35
Lamtor1
HA
TfR
RAB11A/B
Disbindin
Pallidin
RAB7
VPS35
Lamtor1
HA

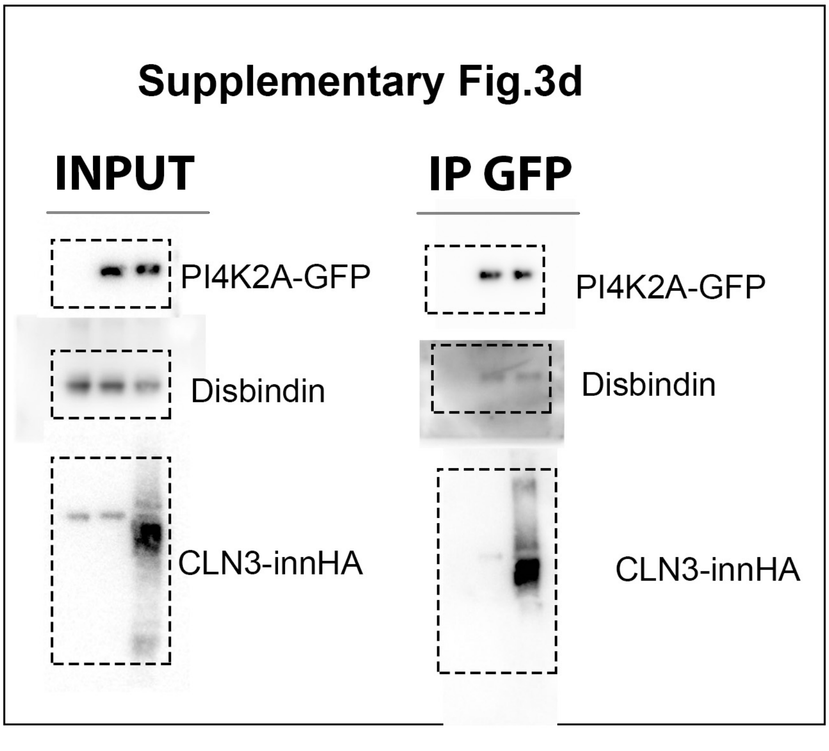
Supplementary Fig.3d
INPUT
IP GFP
PI4K2A-GFP
Disbindin
CLN3-innHA
PI4K2A-GFP
Disbindin
CLN3-innHA

Supplementary Fig.4a

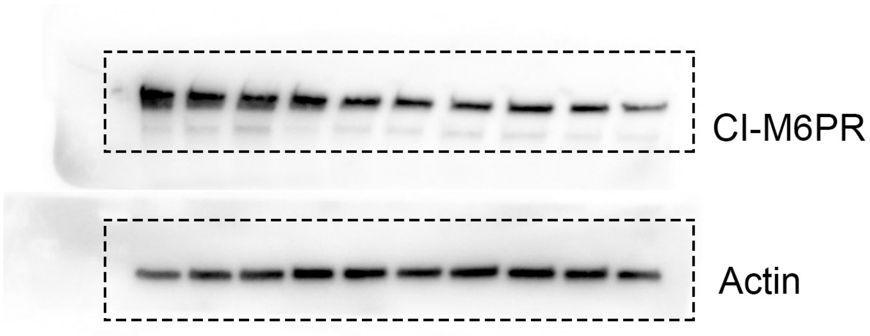

Supplementary Fig.5d

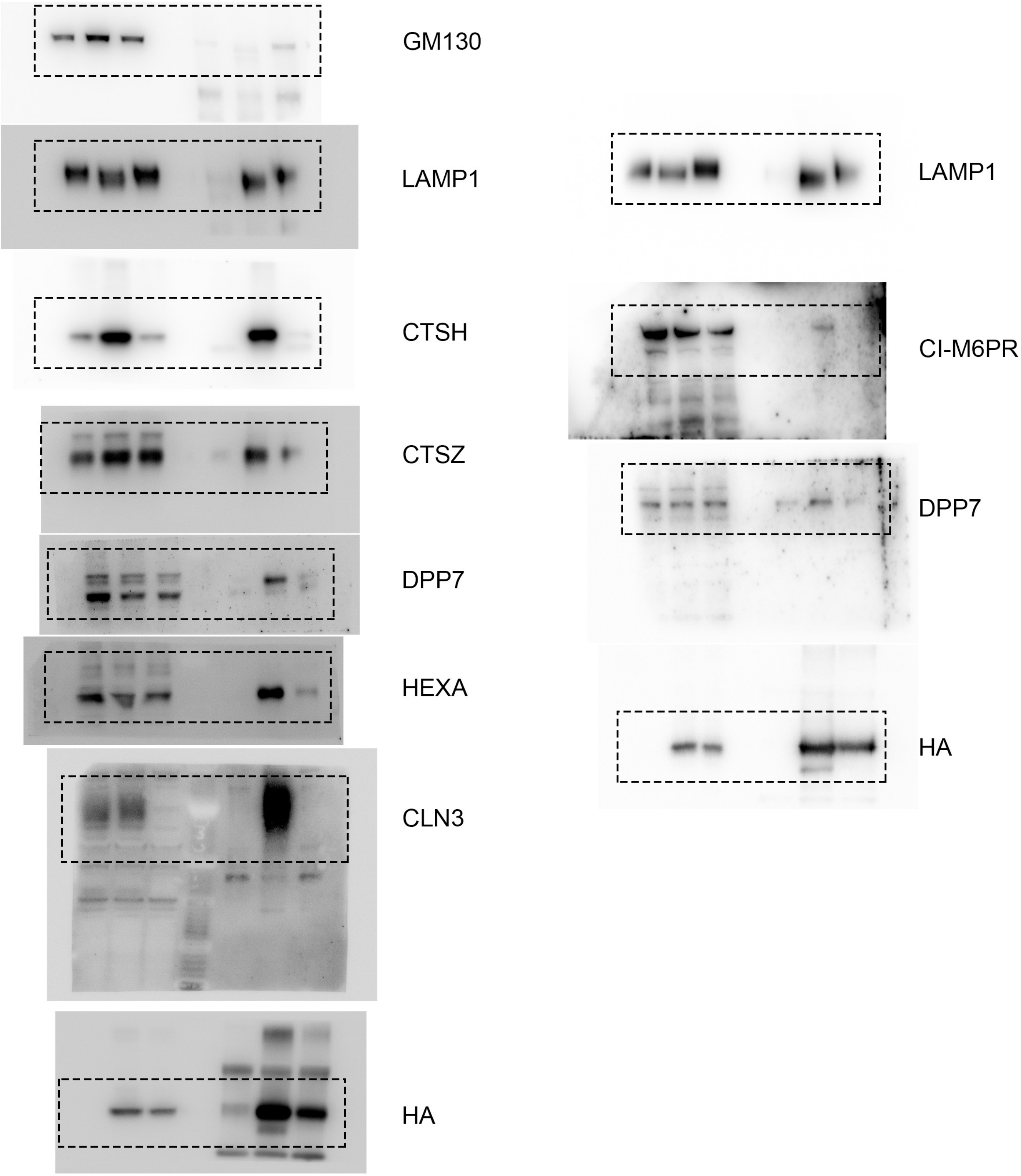

Supplementary Fig.6a

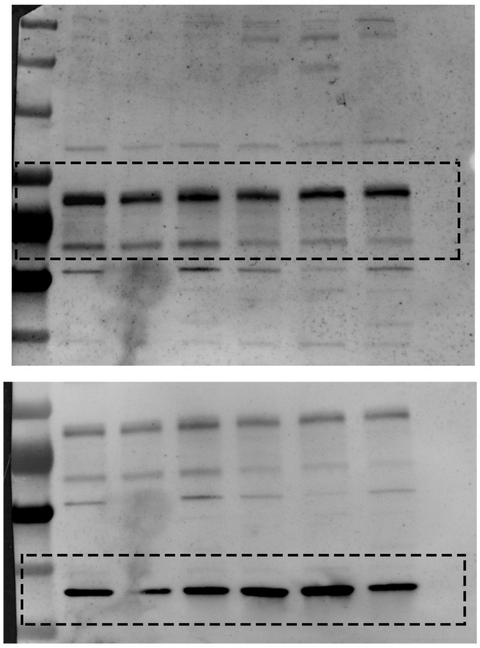

Supplementary Fig.6b

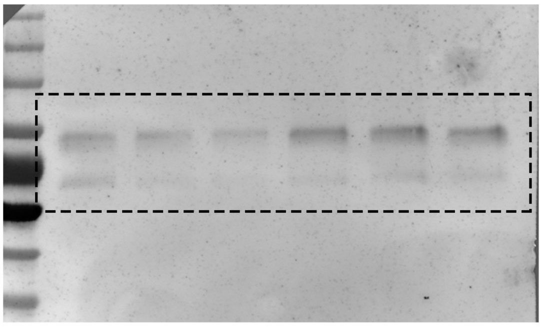

Supplementary Fig.7b

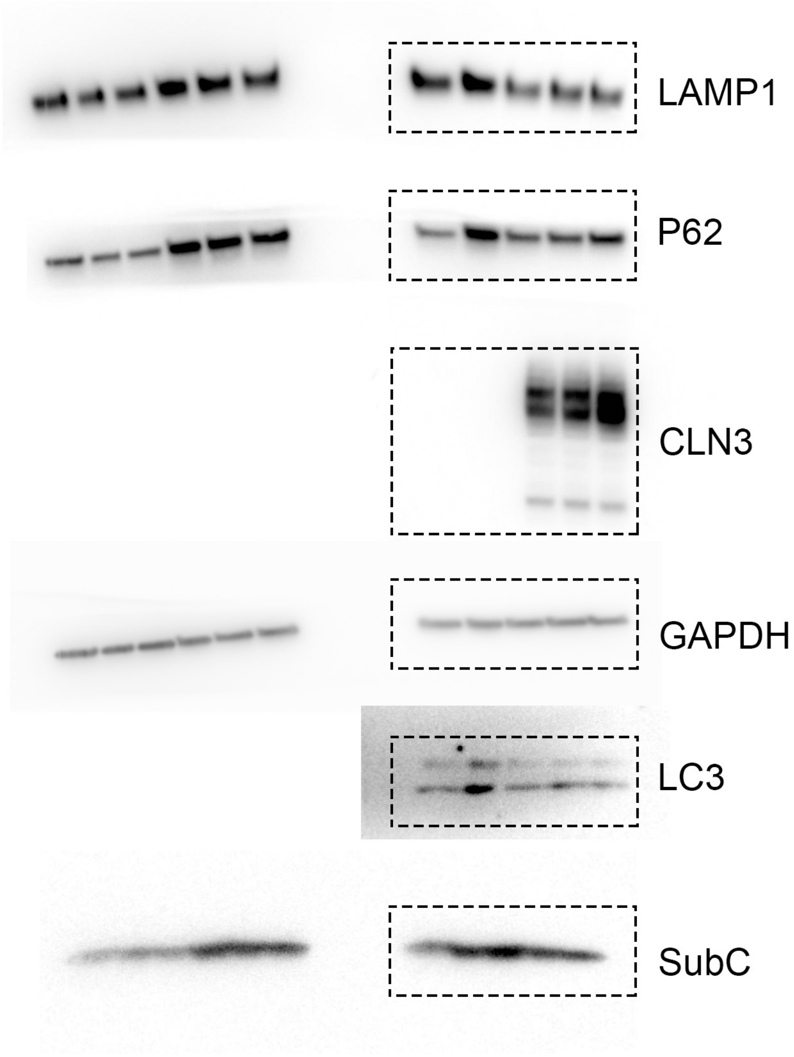

Supplementary Fig.8g

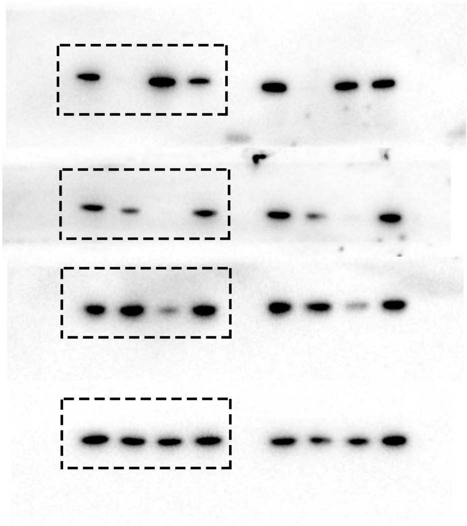

Supplement: Supplementary file 11 — Source Data [file 41467_2023_39643_MOESM11_ESM.zip › source data/Source images blots.pdf]
